# Optimizing health resource allocation for improving timely HIV diagnosis in China

**DOI:** 10.1002/jia2.26221

**Published:** 2024-03-05

**Authors:** Shihao He, Wei Dong, Christopher K. Fairley, Zengbin Li, Yudong Wei, Hao Lai, Rui Li, Pengyi Lu, Mingwang Shen, Zunyou Wu, Lei Zhang

**Affiliations:** ^1^ China‐Australia Joint Research Center for Infectious Diseases School of Public Health Xi'an Jiaotong University Health Science Center Xi'an China; ^2^ National Center for AIDS/STD Control and Prevention (NCAIDS) Chinese Center for Disease Control and Prevention (China CDC) Beijing China; ^3^ Melbourne Sexual Health Centre Alfred Health Melbourne Victoria Australia; ^4^ Central Clinical School Faculty of Medicine Monash University Melbourne Victoria Australia; ^5^ Key Laboratory for Disease Prevention and Control and Health Promotion of Shaanxi Province Xi'an China; ^6^ The Interdisciplinary Center for Mathematics and Life Sciences School of Mathematics and Statistics Xi'an Jiaotong University Xi'an China; ^7^ Key Laboratory of Environment and Genes Related to Diseases (Xi'an Jiaotong University) Ministry of Education Xi'an China

**Keywords:** cost for new case detection, high‐risk and general population, HIV testing, mathematical modelling, optimized resource allocation, timely diagnosis

## Abstract

**Introduction:**

The Joint United Nations Programme on HIV/AIDS (UNAIDS) updated the 95‐95‐95 targets for the HIV endgame in 2030. To achieve the first target in a timely manner, we investigate the optimized strategy of resource allocation to maximize timely HIV diagnosis in 14 populations in China.

**Methods:**

We developed a mathematical model by integrating epidemiological, demographical and behavioural data from 12 high‐risk and two general populations to evaluate the impact of various resource allocation strategies of HIV testing on HIV incidence in China. We identified the optimized allocation strategy that maximizes the number of HIV diagnoses at an estimated total spending on HIV tests in China and calculated the per‐capita cost of new HIV case detection.

**Results:**

We estimated that 144,795 new HIV cases may occur annually in 14 populations in China, with a total annual spending of US$2.8 billion on HIV testing. The largest proportion of spending was allocated to general males (44.0%), followed by general females (42.6%) and pregnant women (5.1%). Despite this allocation strategy, only 45.5% (65,867/144,795, timely diagnosis rate) of annual new infections were diagnosed within a year of acquisition, with a cost of $42,852 required for each new HIV case detection. By optimizing the allocation of HIV testing resources within the same spending amount, we found that general females received the highest proportion of spending allocation (45.1%), followed by low‐risk men who have sex with men (13.9%) and pregnant women (8.4%). In contrast, the proportion of spending allocation for the general males decreased to 0.2%. With this optimized strategy, we estimated that 120,755 (83.4%) of annual new infections would be diagnosed within a year of acquisition, with the cost required for one HIV case detection reduced to $23,364/case. Further spending increases could allow for significant increases in HIV testing among lower‐risk populations.

**Conclusions:**

Optimizing resource allocation for HIV testing in high‐risk populations would improve HIV timely diagnosis rate of new infections and reduce cost per HIV case detection.

## 1 INTRODUCTION

The Joint United Nations Programme on HIV/AIDS (UNAIDS) estimated that 39 million people were living with HIV globally in 2022 [1]. In China, the estimated number of people living with HIV doubled from 650,000 in 2005 to 1,116,000 in 2021 [2, 3]. The proportion of annually diagnosed cases via sharing of drug injecting equipment and mother‐to‐child transmission (MTCT) decreased from 44.2% and 1.5% in 2005 to 0.6% and 0.2% in 2021, respectively, since the national methadone maintenance treatment and prevention of maternal‐to‐child‐transmission (PMTCT) programme were launched [3]. Conversely, the proportion of sexual transmission increased from 11.6% in 2005 to 97.9% in 2021 due to rising commercial and unprotected sexual activities [3, 4].

The UNAIDS has proposed an ambitious global target of 95‐95‐95 (95% of people living with HIV are aware of their HIV status, 95% of those aware of their status initiate treatment and 95% of those on treatment achieve viral suppression) by 2025 to end the HIV epidemic in 2030 [1]. In China, the progress towards this target was 79%, 93% and 96% [5] by the end of 2020. This progress is a result of substantial funding from the Chinese government for HIV/AIDS prevention, diagnosis and treatment over the past two decades, for example in reducing the HIV prevalence among people who inject drugs (PWID) from 7.5% in 2005 to 2% in 2018 [6]. In 2021, the Chinese government and individuals spent approximately $1.8 billion US dollars (USD) on direct HIV testing services. The overall person‐times of HIV testing increased to 322.8 million for people (age 15+) and newborns, nearly six‐fold the 55.6 million in 2009 [7, 8]. However, the disparity of the low first 95% target remains compared with other upper‐middle‐income countries (e.g. 96% in Botswana and 94% in South Africa) [1]. Therefore, intensifying efforts towards HIV testing programmes is critical to improve diagnosis in China.

Timely HIV diagnosis, defined as within 12 months of acquisition [9], is crucial for detecting more infections and further treatment [10]. To achieve timely diagnosis and subsequent care, scaling up HIV screening is of utmost importance in China. Since 2003, the national HIV sentinel surveillance (HSS) network has substantially expanded the accessibility to HIV testing, especially for populations at risk. HIV testing coverage among PWID increased from less than 10% to 41% from 2004 to 2017 [11, 12]. Further, targeted HIV screening for men who have sex with men (MSM) also saw a three‐fold increase from 21% in 2007 to 60% in 2021 [3, 13]. As of 2020, the HSS has covered >1800 healthcare or community‐based sites in 27 Chinese provinces and monitored 12 high‐risk populations (e.g. MSM, PWID) [14, 15, 16], providing accessible HIV testing to each population. However, the accessibility to HIV testing varies across populations, depending on their willingness to test. In particular, 65% of MSM and 63% of money boys (MB) are willing to undergo HIV testing due to improved privacy protection at community‐based HIV testing sites [17, 18]. In contrast, willingness among transgender women and PWID is relatively lower (48% and 45%) due to social stigma, discrimination and injecting drug use compared with MSM [19, 20, 21, 22, 23]. Lower accessibility and willingness often means more efforts and costs are necessary to link these populations to HIV testing and care services.

Overall, HIV testing rates among low‐risk populations are increasing in China. Mandatory pre‐surgical HIV testing and voluntary pre‐marital HIV testing have been widely accepted [24]. However, most of these testing programmes are driven by individual willingness and are not part of a coordinated effort. This lack of coordination due to low accessibility to tests in high‐risk populations may lead to disparities in HIV testing rates and hinder progress towards timely diagnosis and treatment. To optimize resource allocation for HIV testing, considering the disease burden, risk of infection and accessibility to various populations is crucial.

Mathematical modelling effectively identifies the optimized resource allocation for HIV interventions in different settings [25, 26, 27, 28, 29]. For example, in developing settings, such as sub‐Saharan Africa, McGillen et al. [25] used a mathematical model to guide domestic and international funders in understanding local HIV epidemics and relevant drivers for HIV prevention. In a developed setting like the United States, Yaylali et al. [26] developed an economic model to determine the most effective way to allocate government funds across HIV‐related interventions and populations at risk (MSM, PWID and heterosexuals) to prevent the maximal number of new cases annually. This paper aims to develop a mathematical model to determine the optimized allocation of health resources for HIV testing across 14 populations in China.

## 2 METHODS

### 2.1 Definition of population groups

We included 14 populations in this study, of which six populations from HSS, including female sex workers (FSW), long‐distancing truck drivers (LDTD), PWID, MSM, male sexual transmission infections clinic attendees (male STI) and pregnant women [30]. MSM and FSW were further stratified into high‐ and low‐risk subgroups (see details in Supplementary Appendix). Based on the Chinese guideline of avoiding spouse‐to‐spouse transmission [31], we included HIV‐serodiscordant couples and divided them into HIV‐negative male partners (NMP) of serodiscordant couples and HIV‐negative female partners (NFP) of serodiscordant couples. MB and transgender women, with a high HIV prevalence of 11.5% in 2019 [32] and 11.2% in 2021 [33], were included in our study. General males and females 15–65 years of age with sexual activities are also included.

### 2.2 Definition of timely diagnosis

People diagnosed with HIV will receive CD4 count testing before treatment, and this individual was regarded as infected by HIV within the last 12 months if the CD4 count > 700/μl [34]. Following a recent study in China, we defined the rate of timely diagnosis as the proportion of new HIV infections diagnosed within 12 months of acquisition [9].

### 2.3 Data sources

In China, sexual contact accounted for 97.9% of new HIV infections in 2021, whereas injecting drug use accounted for only 0.6%. Therefore, we only simulated HIV transmission via sexual contact [3]. We obtained the following data (Tables S1–S7) for each population from published literature or websites as input parameters of the model and used an optimization algorithm to conduct analysis with the baseline year 2021: (1) the demographic and sexual behavioural data, including population size, HIV prevalence, condom use in the last sexual behaviour and the frequency of sexual behaviours (both with and without a condom) over the past year; (2) the HIV testing and treatment data, including HIV testing rate over the past 12 months, antiretroviral therapy (ART) coverage rate and willingness to test. We estimated the distribution of sexual behaviours for each population and the rate of condom use per sex contact (Figures S1 and S2). Further, we collated HIV testing‐related costs (cost units were USD in 2021) data: the cost of HIV antibody screening (enzyme‐linked immunosorbent assay, ELISA) was $5.6 [7], and the cost of HIV antibody confirmatory test (western blot, WB) was $52.3 [7]. We also estimated the cost (USD) for the linkage to care for each population (Table S8 see detail in Supplementary Appendix) [35]. Costs were not discounted, as we only simulated new HIV infections and test resource allocation within 1 year. All data were collated between June 2021 and December 2022.

### 2.4 Model development

We developed a mathematical model to optimize the spending allocation of each population. We defined the optimized resource allocation outcome as the number of HIV diagnoses maximized at the estimated total testing cost for 14 populations within 1 year. All analyses and simulations were performed in MATLAB R 2023a. This model was constructed in three steps (Formulas 1–6).

First, we estimated the number of new HIV infections acquired via sexual transmission between population groups ($I_{j})$. The estimation was based on “the force of infection” $\lambda_{ij}$ [36], (*i,j* = 1, 2, ⋯, 14, representing 14 population groups), with the *i‐th* population infecting the *j‐th* population over 1 year, as shown in Equations (1) and (2). The annual HIV incidence rate $R_{i}$ (Equation 3) for *i‐th* population is calculated by using new infections divided by population size, *i* = 1,2, ⋯, 14.

(1) $$\lambda_{ij}=p_{i}\left(1-p_{j}\right)N_{j}\beta_{ij}n_{i}m_{ij}\left(1-\varepsilon_{ART}\theta_{i}^{ART}\right)\left(1-\varepsilon_{C}\theta_{ij}^{C}\right)$$

(2) $$I_{j}=\sum_{i=1}^{14}\lambda_{ij}$$

(3) $$R_{i}=\sum_{i=1}^{14}I_{i}/N_{i}$$

where the HIV prevalence and population size of the *i* population are $p_{i}$ and $N_{i}$, respectively, and the average number of sexual acts per year are $n_{i}$. The transmission probability of per sexual contact between the *i* and *j* population is $\beta_{ij}$, and the condom usage rate and the distribution of sexual behaviors are $\theta_{ij}^{C}$ and $m_{ij}$, respectively. $\theta_{i}^{ART}$ is the ART coverage rate among the population *i*. The effectiveness of ART ($\varepsilon_{ART}$) and the efficacy of condoms ($\varepsilon_{C}$) in decreasing the risk of HIV infection was 85% and 90% [37, 38, 39], respectively.

Second, we defined the total cost (C) for HIV testing as the sum of linkage to care cost, HIV antibody screening cost and diagnosis confirmation cost based on the population‐specific testing rate $t_{i}$, *i* = 1,2, ⋯, 14 (Equation 4).

(4) $$\begin{array}{ccc} C & = & \left(c_{i}+c_{1}\right)\sum_{i=1}^{14}t_{i}N_{i}\left(1-p_{i}\right)+c_{2}\sum_{i=1}^{14}t_{i}I_{i}+c_{2}\sum_{i=1}^{14}t_{i}N_{i}p_{i}\left(1-\alpha_{d}\right)\\ & & +c_{2}\sum_{i=1}^{14}t_{i}N_{i}\left(1-p_{i}\right)\left(1-T_{spc}\right) \end{array}$$

where the cost of the linkage to care for *i* population was $c_{i}$. The cost of the HIV antibody screening was *c*_1_. The cost of the HIV antibody confirmatory test was *c*_2_. The fraction of people living with HIV who know their HIV status $\alpha_{d}$ was 79% [5]. The specificity of HIV antibody screening $T_{spc}$. was 99% [40]. People who are initially screened positive are retested to confirm the diagnosis of HIV infection or to exclude false positives.

Third, we estimated two outcome indicators to compare the effectiveness of HIV testing before and after optimizing resource allocation: (a) the timely diagnosis rate θ among annual new infections (Equation 5), and (b) the cost (Equation 6) of detecting one infection case ($C_{d}$).

(5) $$\theta =\sum_{i=1}^{14}t_{i}I_{i}/\sum_{i=1}^{14}I_{i}$$

(6) $$C_{d}=C/\sum_{i=1}^{14}t_{i}I_{i}$$

### 2.5 Model calibration

We collated the reported HIV incidence rates of 14 populations from published literature (Table S9). The model was calibrated using the nonlinear least‐squares method, as illustrated in Figure S3a, by comparing the estimated incidence rates with the reported incidence rates. The calibration procedure is illustrated in the Supplementary Appendix.

### 2.6 Model optimization

We estimated the baseline annual spending on HIV testing (C) in China to be $2.8 billion ($1.5 billion for linkage to care and $1.3 billion for HIV screening and confirmatory test) based on Equation (4). Routine HIV screening among pregnant women is primarily conducted through PMTCT programmes for HIV, syphilis and hepatitis B and had achieved a coverage of 97.5% in 2021 [41]. To optimize resource allocation of HIV testing, we allowed the HIV testing rate of each population ($x_{i}$, *i* = 1,2 ⋯, 14) to vary between 0% and 100% except for pregnant women, with a range of 97.5% and 100%. In this case, we assumed the designated cost for PMTCT cannot be reallocated to other populations and is not included in allocation optimization. We hypothesized that each 1% increase in $x_{i}$ would result in a corresponding marginal cost $\left(q_{i}\right)$ in $c_{i}$ ( $q_{i}=\;1\%/\left(1-x_{i}\right)$) (see details in Supplementary Appendix). We presumed the relationship between testing rate and linkage to care cost followed an inversely proportional function. Subsequently, we employed three parameters ($q_{i}$, $g_{i}$ and $h_{i}$) to establish a fitted linkage to care cost for the *i‐th* population after optimization (Figure S4 and Table S10), denoted as ${c_{i}}^{\prime }$(Equation 7, details in Supplementary Appendix).

(7) $${c_{i}}^{\prime }=q_{i}/\left(x_{i}+g_{i}\right)+h_{i}$$

We defined the HIV testing rates at optimized resource allocation to be $x_{i}^{\prime }$ and the optimized total cost of HIV testing to be $C^{\prime }$ (Equation 8).

(8) $$\begin{array}{ccc} C^{\prime } & = & \left({c_{i}}^{\prime }+c_{1}\right)\sum_{i=1}^{14}{x_{i}}^{\prime }N_{i}\left(1-p_{i}\right)+c_{2}\sum_{i=1}^{14}{x_{i}}^{\prime }I_{i}+c_{2}\sum_{i=1}^{14}{x_{i}}^{\prime }N_{i}p_{i}\left(1-\alpha_{d}\right)\\ & & +c_{2}\sum_{i=1}^{14}{x_{i}}^{\prime }N_{i}\left(1-p_{i}\right)\left(1-T_{spc}\right) \end{array}$$

The optimization of $x_{i}$ was performed to simultaneously meet the following two conditions: (1) the model output was considered mathematically optimized when the objective function for the number of HIV diagnoses was maximized by the *fmincon* algorithm in MATLAB (Equation 9), and (2) subjected to the condition that the optimized total cost cannot exceed the latest reported total cost (Equation 10).

(9) $$\max\left(\sum_{i=1}^{14}{x^{\prime }}_{i}I_{i}\right)$$

(10) $$C^{\prime }\leq C$$

To obtain the median and interquartile range of the optimized testing rate, we obtained a sampling interval by multiplying the baseline demographic and sexual behavioural data by 0.75 and 1.25 times, respectively. We used Latin square hypercube sampling to generate 1000 random samples of each parameter value in the sampling interval and calculated 1000 optimized testing rates.

### 2.7 Sensitivity analysis

Sensitivity analyses included two components. First, we examined the impact of varying the spending allocated for HIV testing at 25%, 50%, five‐fold and 10‐fold increments of the estimated baseline annual cost. This was done to evaluate the potential changes in resource allocation resulting from different spending constraints. Second, we repeated the model simulation by allowing the testing rate for pregnant women to vary between 0% and 100%, which was the same as in the other 13 populations.

## 3 RESULTS

### 3.1 HIV testing rates in 14 populations under the baseline allocation

We estimated the baseline annual spending on HIV testing to be $2.8 billion for the 14 populations. The proportion of resource allocation for HIV testing is the highest in general males (44.0%), followed by general females (42.6%) and pregnant women (5.1%). Spending in these three populations accounted for over 90% of the total spending (Table 1). With this spending, the HIV testing rate over the past 12 months was the highest among pregnant women (97.5%), followed by serodiscordant couples (82.7%) and high‐risk MSM (60.0%) (Table 1).

**Table 1:** The cost of detecting one infection case and diagnoses number for each population in baseline and optimized HIV testing strategy

			Baseline testing strategy				Optimized testing strategy			
Population	Estimated new infections in 12 months	Incidence rate (per 10 thousand)	Baseline testing rate (%)	Diagnoses of new infection	Cost of detecting one HIV case (USD)	Allocation of HIV resources (%)	Optimized testing rate (%) (median, IQR)	Diagnoses of new infection (median, IQR)	Cost of detecting one HIV case (USD) (median, IQR)	Allocation of HIV resources (%)
Transgender women	11,743 (8968–15,451)	141.5 (108.1–186.2)	34.6	4063 (3103–5346)	996 (757–1305)	0.1	98.7 (98.6–98.7)	11,590 (8847–15,256)	4143 (3162–5457)	1.7 (1.7–1.8)
High‐risk MSM	31,962 (24,289–42,228)	88.8 (67.5–117.3)	60.0	19,177 (14,573–25,337)	1356 (1026–1784)	0.9	98.4 (98.3–98.4)	31,434 (23,869–41,564)	5582 (4275–7207)	6.2 (5.9–6.4)
Money boys	5798 (4437–7568)	72.5 (55.5–94.6)	32.0	1855 (1420–2422)	1671 (1280–2183)	0.1	95.6 (95.4–95.8)	5545 (4235–7252)	12,848 (9806–16,962)	2.5 (2.4–2.6)
Low‐risk MSM	28,422 (21,098–38,175)	33.8 (25.1–45.4)	60.0	17,053 (12,659–22,905)	3483 (2593–4692)	2.1	95.6 (95.2–95.9)	27,170 (20,086–36,616)	14,669 (11,126–19,252)	13.9 (13.0–15.0)
PWID	22,652 (17,403–29,588)	105.5 (81.0–137.7)	59.0	13,365 (10,268–17,457)	1391 (1065–1810)	0.7	97.3 (97.2–97.4)	22,042 (16,922–28,822)	8097 (6112–10,504)	6.3 (6.1–6.5)
High‐risk FSW	1379 (1027–1849)	3.7 (2.8–5.0)	60.0	827 (616–1109)	30,246 (22,561–40,604)	0.9	85.8 (85.0–86.5)	1183 (873–1599)	56,507 (42,685–74,878)	2.4 (2.3–2.5)
Low‐risk FSW	1624 (1209–2177)	3.8 (2.8–5.0)	60.0	974 (726–1306)	30,153 (22,489–40,490)	1.0	78.3 (77.0–79.3)	1271 (931–1727)	74,135 (55,650–97,428)	3.3 (3.1–3.5)
NMP	676 (501–908)	48.3 (35.8–64.8)	82.7	559 (415–751)	2216 (1650–2989)	0.0	96.5 (96.1–96.7)	652 (482–878)	10,900 (8112–14,418)	0.3 (0.2–0.3)
NFP	500 (370–672)	10.2 (7.6–13.7)	82.7	414 (306–556)	10,292 (7655–13,902)	0.2	92.3 (91.7–92.8)	462 (339–624)	28,142 (21,186–38,017)	0.5 (0.4–0.5)
Male STI	14,487 (11,564–18,524)	24.1 (19.3–30.9)	12.5	1811 (1446–2315)	5000 (3911–6264)	0.3	94.0 (93.7–94.5)	13,621 (10,836–17,503)	19,296 (15,011–24,583)	9.2 (8.7–9.8)
LDTD	212 (159–278)	0.1 (0.1–0.1)	20.7	44 (33–57)	1,198,893 (915,114–1,600,556)	1.9	0.2 (0.1–0.3)	0 (0–1)	1,086,277 (821,999–1,445,547)	0.0 (0.0–0.0)
General males	3004 (2380–3944)	0.1 (0.1–0.1)	20.6	619 (491–813)	2,005,013 (1,527,140–2,530,224)	44.0	0.1 (0.1–0.1)	3 (2–4)	1,644,257 (1,271,241–2,059,581)	0.2 (0.2–0.2)
Pregnant women	649 (481–888)	0.4 (0.3–0.6)	97.5	633 (469–866)	228,746 (167,103–308,817)	5.1	97.5 (97.5–97.5)	633 (469–866)	371,709 (277,457–501,060)	8.4 (8.4–8.4)
General females	21,687 (16,268–29,587)	0.5 (0.4–0.7)	20.6	4472 (3354–6101)	269,046 (197,208–358,671)	42.6	24.0 (22.8–25.1)	5214 (3707–7427)	241,813 (180,646–324,955)	45.1 (42.2–47.3)
Overall	144,795 (110,154–191,837)	1.5 (1.1–2.0)	45.5^a^	65,867 (49,877–87,342)	42,852 (32,316–56,590)	100	83.4^a^ (82.3–84.3)	120,755 (90,640–161,798)	23,364 (18,231–30,624)	100

Abbreviations: FSW, female sex workers; IQR, interquartile range; LDTD, long‐distancing truck drivers; LMSM, men who have sex with men; Male STI, male sexual transmission infections clinic attendees; MSM, men who have sex with men; NFP, HIV‐negative female of serodiscordant couples; NMP, HIV‐negative male of serodiscordant couples; PWID, people who inject drugs.

^a^ represents the timely diagnosis rate among total annual new infections.

### 3.2 HIV timely diagnosis and cost for HIV case detection in 14 populations under the baseline allocation

**Figure 1:**
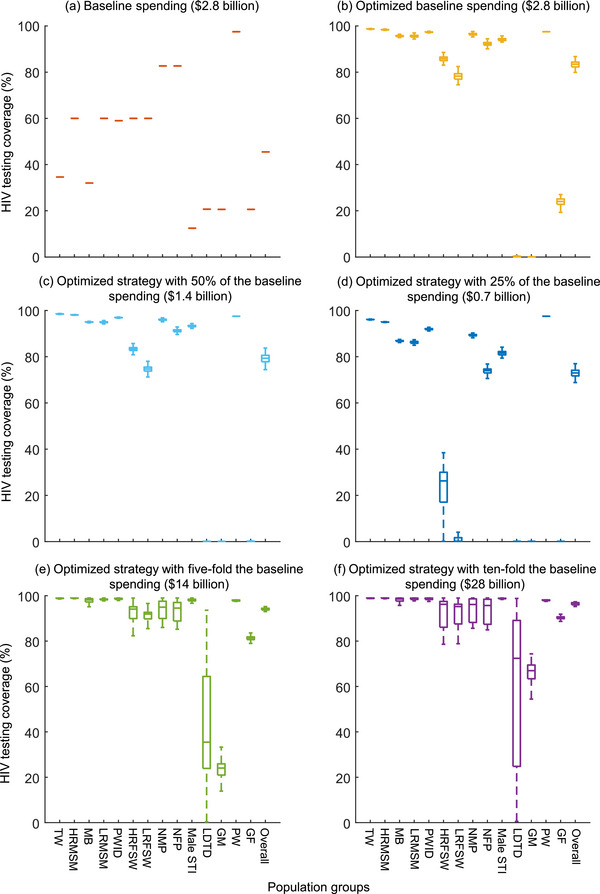
HIV testing coverage in 14 Chinese populations at baseline and five scenarios of resource allocations. GF, general females; GM, general males; HRFSW, high‐risk female sex workers; HRMSM, high‐risk men who have sex with men; LDTD, long‐distancing truck drivers; LRFSW, low‐risk female sex workers; LRMSM, low‐risk men who have sex with men; Male STI, male sexual transmission infections clinic attendees; MB, money boys; NFP, HIV‐negative female partners of serodiscordant couples; NMP, HIV‐negative male partners of serodiscordant couples; PWID, people who inject drugs; PW, pregnant women; TW, transgender women. Overall represents the proportion of detecting among total annual new infections.

**Figure 2:**
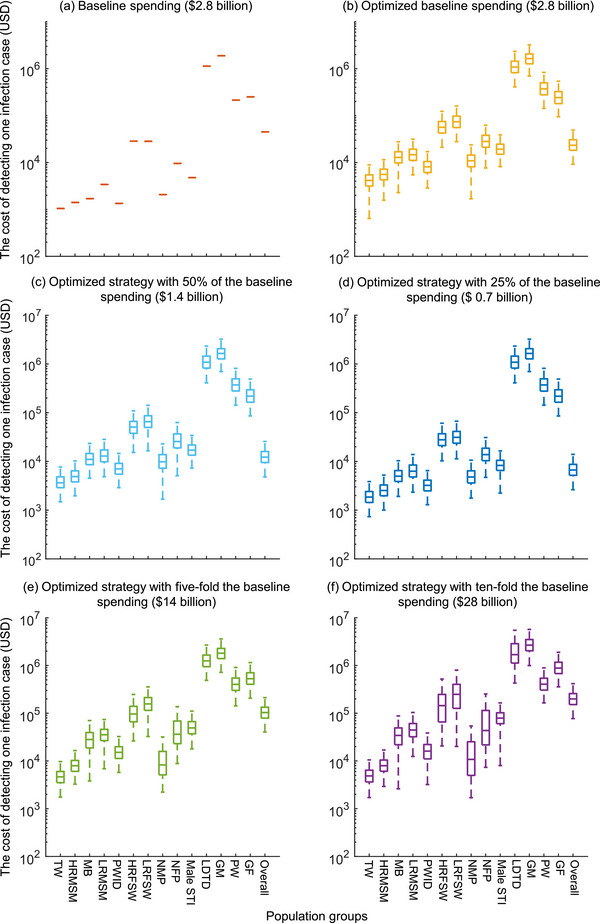
The cost of detecting one HIV‐infection case under baseline and five scenarios of different total costs. GF, general females; GM, general males; HRFSW, high‐risk female sex workers; HRMSM, high‐risk men who have sex with men; LDTD, long‐distancing truck drivers; LRFSW, low‐risk female sex workers; LRMSM, low‐risk men who have sex with men; Male STI, male sexual transmission infections clinic attendees; MB, money boys; NFP, HIV‐negative female partners of serodiscordant couples; NMP, HIV‐negative male partners of serodiscordant couples; PWID, people who inject drugs; PW, pregnant women; TW, transgender women. Overall represents the cost of detecting one infection case among whole population groups.

We estimated the total number of annual new HIV infections would be 144,795 (interquartile range [IQR]: 110,154–191,837) cases across 14 population groups in 2021 (Table 1). With the baseline resource allocation for HIV testing across these population groups, an estimated 65,867 (45.5%) new infections would be diagnosed within 12 months. In particular, the largest number of new infections would be diagnosed in high‐risk MSM (60.0% = 19,177/31,962, Figure 1a and Table 1), followed by low‐risk MSM (60.0% = 17,053/28,422) and PWID (59.0% = 13,365/22,652). Across all 14 Chinese populations, the average cost for HIV case detection was $42,852/case (Figure 2a and Table 1). The cost for HIV case detection in each population is listed in Table 1.

### 3.3 HIV testing rates among 14 populations under the optimized allocation

**Figure 3:**
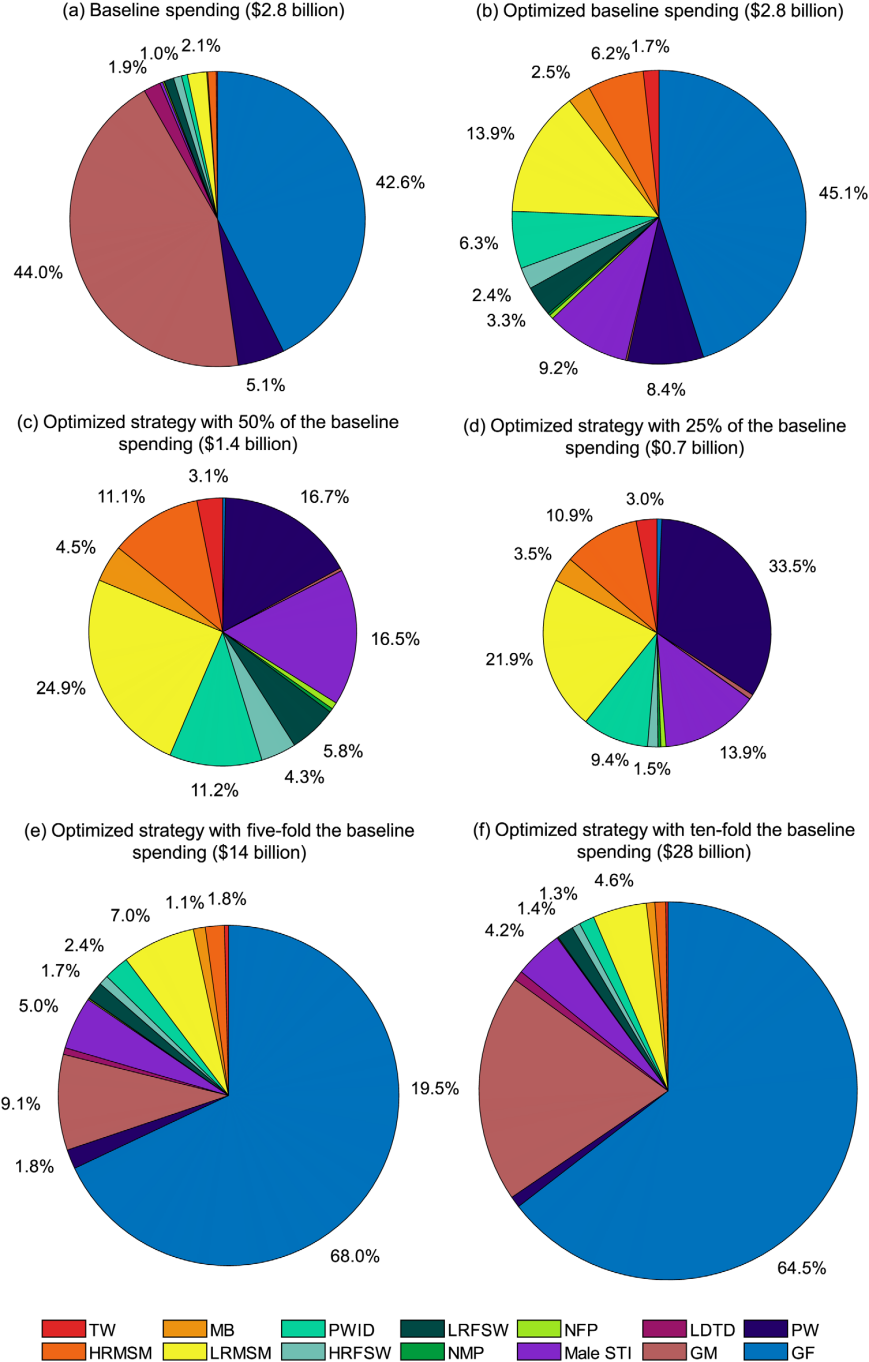
The proportion of spending for HIV testing among 14 populations under baseline and five scenarios of different total costs. The proportion of spending < 1% was not labeled in the pie plot. GF, general females; GM, general males; HRFSW, high‐risk female sex workers; HRMSM, high‐risk men who have sex with men; LDTD, long‐distancing truck drivers; LRFSW, low‐risk female sex workers; LRMSM, low‐risk men who have sex with men; Male STI, male sexual transmission infections clinic attendees; MB, money boys; NFP, HIV‐negative female partners of serodiscordant couples; NMP, HIV‐negative male partners of serodiscordant couples; PWID, people who inject drugs; PW, pregnant women; TW, transgender women. Overall represents the cost of detecting one infection case among whole population groups.

We optimized the resource allocation for HIV testing across the populations by relocating resources to minimize the cost of HIV case detection without changing the total investment. With the optimized strategy, more funding should be allocated to high‐risk populations. In particular, the proportion of resource allocation for HIV testing is the highest in general females (45.1%), followed by low‐risk MSM (13.9%) and male STI (9.2%) (Figure 3b). With the optimized strategy, the HIV testing rate among transgender women would increase to 98.7%, followed by 98.4% in high‐risk MSM and 97.5% in pregnant women.

### 3.4 HIV timely diagnoses and cost for HIV case detection among 14 populations under the optimized allocation

We found that the optimized strategy would substantially increase the number of HIV cases detected. With the optimized strategy, the number of HIV diagnoses within 12 months of infection would increase to 120,755 (83.4% of annual new infections). In particular, the largest number of diagnoses would occur in high‐risk MSM (98.4% = 31,434/31,962, Figure 1b and Table 1), followed by low‐risk MSM (95.6% = 27,170/28,422) and PWID (97.4% = 22,042/22,652). The overall cost for HIV case detection across the 14 populations was $23,364/case (Figure 2b and Table 1).

### 3.5 Impact of spending increase on the optimized allocation

If the spending for HIV testing would increase five‐fold the baseline spending, the proportion of resource allocation for general females would change to 68.0% (Figure 3e and Table 2). The overall timely diagnosis rate in annual new HIV infections would increase to 94.1% (Figure 1e and Table 2). Further, if the spending for HIV testing increased 10‐fold, the spending allocation for general females would change to 64.5% (Figure 3f and Table 2). The timely diagnosis rate would increase to 96.7% (Figure 1f and Table 2).

**Table 2:** The cost of detecting one infection case and diagnoses number for each population at two scenarios of increased estimated baseline spending

	Optimized strategy with five‐fold baseline spending				Optimized strategy with 10‐fold baseline spending			
Population	Optimized testing rate (%) (median, IQR)	Diagnoses of new infection (median, IQR)	Cost of detecting one HIV case (USD) (median, IQR)	Allocation of HIV resources (%)	Optimized testing rate (%) (median, IQR)	Diagnoses of new infection (median, IQR)	Cost of detecting one HIV case (USD) (median, IQR)	Allocation of HIV resources (%)
Transgender women	98.9 (98.8–99.0)	11,608 (8859–15,289)	4648 (3520–6021)	0.4 (0.4–0.4)	98.9 (98.8–99.0)	11,616 (8858–15,294)	4870 (3638–6407)	0.2 (0.2–0.2)
High‐risk MSM	98.9 (98.9–99.0)	31,620 (24,011–41,796)	7949 (6070–10,257)	1.8 (1.7–1.9)	99.0 (98.8–99.0)	31,628 (24,009–41,804)	7971 (6089–10,452)	0.9 (0.9–0.9)
Money boys	98.2 (97.2–98.7)	5691 (4314–7470)	28,184 (18,766–39,420)	1.1 (0.8–1.6)	98.5 (97.6–98.9)	5714 (4332–7485)	34,328 (21,929–49,252)	0.7 (0.5–1.0)
Low‐risk MSM	98.3 (98.2–98.5)	27,953 (20,712–37,610)	35,450 (26,694–46,005)	7.0 (6.3–7.7)	98.8 (98.3–98.9)	28,092 (20,749–37,774)	44,561 (32,147–60,924)	4.6 (3.5–5.4)
PWID	98.7 (98.4–98.9)	22,348 (17,125–29,252)	15,189 (11,292–19,884)	2.4 (2.1–2.8)	98.8 (98.3–99.0)	22,385 (17,111–29,279)	16,085 (11,065–22,109)	1.3 (1.0–1.5)
High‐risk FSW	94.1 (89.9–95.1)	1297 (924–1759)	96,359 (66,080–139,563)	0.9 (0.6–1.2)	96.2 (86.1–97.5)	1326 (884–1802)	144,596 (65,689–248,146)	0.7 (0.2–1.1)
Low‐risk FSW	91.9 (89.8–92.7)	1493 (1086–2018)	156,779 (114,878–212,975)	1.7 (1.4–1.9)	95.2 (87.6–96.2)	1546 (1059–2096)	248,879 (126,384–402,948)	1.4 (0.6–1.9)
NMP	94.9 (90.0–97.6)	642 (451–886)	8256 (5117–15,882)	0.0 (0.0–0.1)	96.1 (88.2–98.6)	650 (442–895)	10,795 (4993–25,248)	0.0 (0.0–0.1)
NFP	94.5 (88.9–97.0)	473 (329–652)	36,271 (23,047–68,827)	0.1 (0.1–0.2)	95.7 (87.4–98.4)	479 (323–662)	43,496 (21,772–114,346)	0.1 (0.0–0.2)
Male STI	97.9 (97.7–98.4)	14,185 (11,301–18,233)	49,272 (37,059–66,724)	5.0 (4.4–6.3)	98.8 (98.7–98.9)	14,308 (11,409–18,316)	79,070 (60,534–102,356)	4.2 (3.9–4.5)
LDTD	35.5 (23.9–64.4)	75 (38–179)	1,249,577 (945,506–1,652,473)	0.6 (0.4–1.3)	72.4 (24.8–89.1)	153 (39–247)	1,690,009 (1,124,323–2,862,949)	0.9 (0.2–1.5)
General males	24.0 (20.9–25.9)	722 (499–1021)	1,810,980 (1,396,110–2,281,648)	9.1 (8.0–10.2)	67.0 (63.4–69.5)	2012 (1509–2739)	2,673,217 (2,034,459–3,497,356)	19.5 (18.1–21.4)
Pregnant women	98.0 (97.7–98.1)	636 (469–872)	402,672 (300,983–546,422)	1.8 (1.7–1.9)	98.1 (97.8–98.2)	637 (470–872)	406,514 (305,483–541,318)	1.0 (0.9–1.0)
General females	81.4 (80.7–81.8)	17,652 (13,122–24,216)	528,785 (404,246–705,454)	68.0 (64.8–69.7)	90.2 (89.9–90.7)	19,572 (14,622–26,827)	882,696 (677,767–1,171,874)	64.5 (59.9–67.4)
Overall	94.1^a^ (93.7–94.5)	136,264 (103,216–181,208)	103,343 (78,932–133,797)	100	96.7^a^ (96.0–96.9)	140,066 (105,753–185,983)	199,296 (151,875–258,014)	100

Abbreviations: FSW, female sex workers; IQR, interquartile range; LDTD, long‐distancing truck drivers; LMSM, men who have sex with men; Male STI, male sexual transmission infections clinic attendees; MSM, men who have sex with men; NFP, HIV‐negative female of serodiscordant couples; NMP, HIV‐negative male of serodiscordant couples; PWID, people who inject drugs.

^a^ represents the timely diagnosis rate among total annual new infections.

### 3.6 Impact of spending decrease on the optimized allocation

If the spending were reduced to 50% of the baseline, the available spending would be allocated mainly to low‐risk MSM (24.9%) (Figure 3c and Table 3). The timely HIV diagnosis rate would reduce to 79.4% (Figure 1c and Table 3). Further, if the spending were reduced to 25% of the baseline spending, the proportion of spending was the highest in pregnant women (33.5%), followed by low‐risk MSM (21.9%) and male STI (13.9%) (Figure 3d and Table 3). The timely HIV diagnosis rate would reduce to 73.0% (Figure 1d and Table 3).

**Table 3:** The cost of detecting one infection case and diagnoses number for each population at two scenarios of decreased estimated baseline spending

	Optimized strategy with 50% of the baseline spending				Optimized strategy with 25% of the baseline spending			
Population	Optimized testing rate (%) (median, IQR)	Diagnoses of new infection (median, IQR)	Cost of detecting one HIV case (USD) (median, IQR)	Allocation of HIV resources (%)	Optimized testing rate (%) (median, IQR)	Diagnoses of new infection (median, IQR)	Cost of detecting one HIV case (USD) (median, IQR)	Allocation of HIV resources (%)
Transgender women	98.5 (98.4–98.5)	11,567 (8828–15,226)	3636 (2894–4833)	3.1 (3.0–3.1)	96.1 (96.0–96.1)	11,284 (8613–14,855)	1855 (1437–2413)	3.0 (2.9–3.0)
High‐risk MSM	98.1 (98.1–98.1)	31,355 (23,816–41,437)	4891 (3795–6406)	11.1 (10.7–11.1)	95.0 (95.0–95.0)	30,361 (23,062–40,134)	2532 (1973–3288)	10.9 (10.8–11.0)
Money boys	95.0 (94.9–95.1)	5509 (4212–7195)	11,020 (8572–14,512)	4.5 (4.3–4.5)	86.8 (86.7–87.0)	5034 (3845–6584)	4986 (3876–6491)	3.5 (3.5–3.6)
Low‐risk MSM	95.0 (94.8–95.1)	27,000 (19,990–36,310)	12,891 (9823–17,216)	24.9 (24.1–25.6)	86.3 (85.8–86.6)	24,533 (18,107–33,064)	6299 (4793–8427)	21.9 (21.4–22.5)
PWID	96.9 (96.9–97.0)	21,955 (16,856–28,694)	7031 (5460–9123)	11.2 (10.8–11.2)	91.9 (91.7–92.2)	20,819 (15,959–27,267)	3229 (2486–4122)	9.4 (9.2–9.6)
High‐risk FSW	83.4 (82.7–83.9)	1150 (850–1552)	50,665 (37,682–66,333)	4.3 (4.1–4.3)	26.3 (17.1–30.0)	362 (175–555)	27,836 (21,089–37,011)	1.5 (0.9–1.7)
Low‐risk FSW	74.7 (73.8–75.5)	1213 (892–1644)	65,366 (49,857–86,703)	5.8 (5.5–5.9)	0.2 (0.1–1.7)	3 (1–37)	31,212 (23,602–41,231)	0.0 (0.0–0.1)
NMP	96.1 (95.8–96.2)	649 (480–873)	9841 (7261–13,777)	0.5 (0.4–0.5)	89.4 (89.1–89.6)	604 (446–814)	4781 (3615–6419)	0.4 (0.4–0.4)
NFP	91.4 (90.8–91.7)	457 (336–616)	25,578 (18,932–36,619)	0.8 (0.8–0.9)	74.0 (73.0–74.7)	370 (270–502)	13,802 (10,364–18,638)	0.7 (0.7–0.7)
Male STI	93.2 (93.0–93.5)	13,508 (10,753–17,325)	17,054 (13,424–21,732)	16.5 (15.8–16.9)	81.6 (80.8–82.2)	11,817 (9344–15,235)	8286 (6524–10,531)	13.9 (13.5–14.3)
LDTD	0.1 (0.1–0.1)	0 (0–0)	1,086,184 (821,698–1,445,347)	0.0 (0.0–0.0)	0.1 (0.1–0.1)	0 (0–0)	1,086,167 (821,689–1,445,344)	0.0 (0.0–0.0)
General males	0.1 (0.1–0.1)	3 (2–4)	1,644,211 (1,271,233–2,059,576)	0.4 (0.4–0.4)	0.1 (0.1–0.1)	3 (2–4)	1,644,208 (1,271,232–2,059,572)	0.7 (0.7–0.7)
Pregnant women	97.5 (97.5–97.5)	633 (469–866)	372,359 (278,597–495,516)	16.7 (16.7–16.7)	97.5 (97.5–97.5)	633 (469–866)	371,448 (277,094–494,874)	33.5 (33.4–33.5)
General females	0.1 (0.1–0.2)	22 (16–54)	218,581 (164,034–294,929)	0.3 (0.3–0.6)	0.1 (0.1–0.1)	22 (16–30)	217,958 (163,878–294,885)	0.7 (0.7–0.7)
Overall	79.4^a^ (77.8–80.6)	114,901 (85,742–154,612)	12,254 (9492–16,011)	100	73.0^a^ (71.7–74.1)	105,669 (78,986–142,214)	6664 (5183–8705)	100

Abbreviations: FSW, female sex workers; IQR, interquartile range; LDTD, long‐distancing truck drivers; LMSM, men who have sex with men; Male STI, male sexual transmission infections clinic attendees; MSM, men who have sex with men; NFP, HIV‐negative female of serodiscordant couples; NMP, HIV‐negative male of serodiscordant couples; PWID, people who inject drugs.

^a^ represents the timely diagnosis rate among total annual new infections.

### 3.7 Impact of removing the constraint of PMTCT on the optimized allocation

We also investigate the scenario where spending for HIV remained unchanged at the baseline level in the absence of the PMTCT programme. In this case, after optimization, the largest proportion of spending for HIV testing would be allocated to general females (49.0%) within the baseline spending (Figure S7 and Table S11). This achieves an 83.6% timely diagnosis rate for annual infections at an overall HIV diagnosis cost of $23,406/case (Figures S5 and S6, Table S11). If the spending for HIV testing would increase five‐fold the baseline spending, the timely HIV diagnosis rate would increase to 94.1% (Table S12). In contrast, the timely HIV diagnosis rate would change to 79.7% if the spending were reduced to 50% of the baseline spending (details in the Supplementary Materials).

## 4 DISCUSSION

Our study investigated the optimized allocation of HIV testing resources to timely diagnose new HIV infections with the baseline spending on HIV testing in China. We estimated that China spends $2.8 billion annually on HIV testing in 14 populations, with over 90% directed to pregnant women and general populations. By optimizing resource allocation, over 55% of spending would shift to high‐risk populations (MSM, PWID, transgender women and male STI), boosting the timely diagnosis rate from 45.5% to 83.4% of the 144,795 new HIV infections. The optimization will also reduce the average cost of HIV case detection from $42,852/case to $23,364/case. Moreover, if the constraint of HIV testing for pregnant women due to the PMTCT programme were removed, the timely diagnosis rate would increase to 83.6%, with a cost of $23,406/case. Additional spending could allow a more convenient resource allocation towards lower‐risk and general populations.

Our study suggests that HIV testing resources should be prioritized towards high‐risk populations. This is consistent with previous modelling studies [42, 43], which advocated prioritizing HIV testing in high‐risk populations (i.e. MSM, PWID and FSW) to effectively scale up diagnosis and treatment programmes and significantly reduce HIV incidence. PMTCT in China has been established as a national programme to prevent vertical transmission of HIV [41], and the triple elimination of mother‐to‐child transmission for HIV, syphilis and hepatitis B virus (HBV) has also decreased the global burden of syphilis and HBV infection recently [44]. To simulate the realistic resource allocation, our model constrained PMTCT resources only to the maternal‐newborn population. We hypothesized five different spending scenarios to prioritize populations for testing based on varying HIV testing spending. Our findings demonstrate that the HIV spending for general males should be prioritized towards high‐risk males (i.e. MSM, MB, PWID, male STI and LDTD) to improve the chance of diagnosing HIV infections. In China, scaling up HIV testing in high‐risk groups like MSM can facilitate timely treatment and reduce further transmission.

In reality, allocating HIV testing resources to high‐risk populations in China may face challenges. Linking certain high‐risk groups (e.g. FSW, MB, PWID and transgender women) to HIV care has been difficult due to the hard reach of these populations. Commercial sex work and injecting drug use are illegal and stigmatized in China, leading sex workers and PWID to avoid health facilities for fear of prosecution. Social stigma and discrimination against transgender women also pose substantial barriers to accessing HIV testing [20]. Our model incorporates an increasing marginal cost of linkage to care for HIV for each incremental increase in the HIV testing rate. We mathematically demonstrate that a certain proportion of the high‐risk population becomes exceedingly difficult to reach, preventing further improvement in the HIV testing rate beyond a plateau. As the yield rate of tests decreases with increasing funding in high‐risk populations, more funds should be allocated to general populations. The Chinese government should prioritize reducing stigma and discrimination to enhance HIV testing willingness among high‐risk populations.

To improve HIV testing and timely diagnosis in China, further spending from the Chinese government and society is crucial. In particular, the Chinese government should consider investing in HSS for timely testing in monitored high‐risk populations. National and private insurance should fully cover HIV testing costs at medical facilities. Additionally, expanding funding for HIV self‐testing kits for community‐based organizations could also effectively increase the testing rate in high‐risk populations.

Our study has several limitations. First, our model only captured sexual transmission routes, accounting for 97.9% of new infections [3], omitting 2.1% of infections transmitted through blood transfusion, mother‐to‐child and sharing of drug injection equipment. Second, we did not distinguish antiretroviral (ARV) drug types, dosage and patient adherence, using average ART effectiveness for simplicity since patients will receive the same free first‐line ARV drugs (e.g. lamivudine and zidovudine) based on Chinese guideline‐recommended treatment regimens [31]. Third, we did not distinguish the difference in condom usage between casual and regular sexual partners due to limited data and used the mean sexual risk for them. Fourth, we did not consider the preventive effect of ART on further generations of infection, as the model only captures HIV incidence in 12 months. The return on investment does not reflect the future benefits and co‐benefits of investing in HIV. Fifth, we used the average frequency of sexual acts rather than the number of sexual partners to calculate the annual infections, as the data on the number of regular, casual and transactional partners for the 14 populations are very limited, and no proxy data were available. This may underestimate the number of sexual acts per year and the number of annual new infections. Sixth, the data on the type of HIV test received by each population group are limited. We assume that each population received the same HIV antibody screening (ELISA) and HIV antibody confirmatory tests (WB). Seventh, due to the limited cost‐related data, we used the latest reported testing coverage in each population and test cost ($5.6 for ELISA and $52.3 for WB) to estimate the total spending on HIV testing services. Finally, given the only available data on the cost of linkage to care among PWID, we projected the cost of linkage to care in this population to inform the other 13 populations.

## 5 CONCLUSIONS

Our study demonstrated that shifting HIV testing resources to high‐risk populations may substantially improve the timely diagnosis of new HIV infections and reduce the cost of case detection. This can be achieved without affecting the baseline HIV PMTCT programme. Spending increase will enable more lower‐risk populations to be tested.

## COMPETING INTERESTS

All authors declare that they have no competing interests.

## AUTHORS’ CONTRIBUTIONS

MS, LZ, ZW and SH conceived and designed the study. MS, SH, WD, ZL, YW, HL, RL and PL collated the data. MS and SH analysed the data, carried out the analysis and performed numerical simulations. SH wrote the first draft of the manuscript. LZ, MS and ZW critically revised the manuscript. All the authors contributed to writing the paper and agreed with the manuscript results and conclusions.

## FUNDING

This work was supported by the National Key R&D Program of China (2022YFC2304900, 2022YFC2505100), the National Natural Science Foundation of China (12171387 [MS], 81950410639 [LZ]); China Postdoctoral Science Foundation (2018M631134 [MS], 2020T130095ZX [MS]); Young Talent Support Program of Shaanxi University Association for Science and Technology (20210307 [MS]); Outstanding Young Scholars Support Program (3111500001 [LZ]); Xi'an Jiaotong University Basic Research and Profession Grant (xtr022019003 [LZ], xzy032020032 [LZ]) and Xi'an Jiaotong University Young Scholar Support Grant (YX6J004 [LZ]); the Bill & Melinda Gates Foundation (20200344 [LZ]). CKF is supported by an Australian NHMRC Leadership Investigator Grant (GNT1172900).

## DISCLAIMER

The funders had no role in study design, data collation and analysis, the decision to publish, or the preparation of the manuscript.

The codes for all model analyses are given on the following website: https://doi.org/10.5281/zenodo.10370698.

## Supporting information


**Figure S1** The distribution of sexual behaviors in each population group.
**Figure S2** The distribution of condom use rate in each population group.
**Figure S3** Model calibration and data fitting based on HIV incidence.
**Figure S4** The relationship between the testing rate and the cost of link‐to‐test among each population
**Figure S5** HIV testing coverage in 14 Chinese populations at baseline and five scenarios of resource allocations without PMTCT
**Figure S6** The cost of detecting one HIV infection case under five scenarios of different total costs without PMTCT
**Figure S7** The proportion of spending for HIV testing among 14 populations under baseline and five scenarios of different total costs without PMTCT
**Table S1** Data source of the latest reported population size
**Table S2** Data source of the HIV prevalence
**Table S3** Data source of HIV testing rate over the past 12 months
**Table S4** Data source of ART coverage rate over the past 12 months
**Table S5** Data source of condom use rate over the last sex
**Table S6** Data source of willingness to test
**Table S7**Data source of frequency of sexual behaviors over the past year
**Table S8**Data source of the cost of linkage to care
**Table S9** HIV incidence in each population for model calibration
**Table S10** The value of three parameters by data fitting
**Table S11** The cost of detecting one infection case and diagnoses number for each population in baseline and optimized HIV testing strategy without PMTCT.
**Table S12** The cost of detecting one infection case and diagnoses number for each population at four different scenarios of resource allocations without PMTCT

## Data Availability

The data that support the findings of this study are available in the Supplementary Materials of this article.
