# Supplementary material for: Optimizing health resource allocation for improving timely HIV diagnosis in China
Source: J Int AIDS Soc. 2024 Mar 5;27(3):e26221. doi: 10.1002/jia2.26221 (PMC10935715; doi:10.1002/jia2.26221)
Supplement: Supplementary file 1 — Figure S1 The distribution of sexual behaviors in each population group. Figure S2 The distribution of condom use rate in each population group. Figure S3 Model calibration and data fitting based on HIV incidence. Figure S4 The relationship between the testing rate and the cost of link‐to‐test among each population Figure S5 HIV testing coverage in 14 Chinese populations at baseline and five scenarios of resource allocations without PMTCT Figure S6 The cost of detecting one HIV infection case under five scenarios of different total costs without PMTCT Figure S7 The proportion of spending for HIV testing among 14 populations under baseline and five scenarios of different total costs without PMTCT Table S1 Data source of the latest reported population size Table S2 Data source of the HIV prevalence Table S3 Data source of HIV testing rate over the past 12 months Table S4 Data source of ART coverage rate over the past 12 months Table S5 Data source of condom use rate over the last sex Table S6 Data source of willingness to test Table S7Data source of frequency of sexual behaviors over the past year Table S8Data source of the cost of linkage to care Table S9 HIV incidence in each population for model calibration Table S10 The value of three parameters by data fitting Table S11 The cost of detecting one infection case and diagnoses number for each population in baseline and optimized HIV testing strategy without PMTCT. Table S12 The cost of detecting one infection case and diagnoses number for each population at four different scenarios of resource allocations without PMTCT [file JIA2-27-e26221-s001.docx]

**Supplementary appendix**

**Optimizing health resource allocation for improving timely HIV diagnosis in China**

This supplementary document describes the definition of populations, details for data collation, model calibration, and estimation of parameters presented in the main text. The timeframe for analysis of this study was 2021, and the cost units were US dollars (USD) in 2021.

***Definition of the targeted population***

We defined the high-risk population as those with HIV-related risk behavior monitored by the China HIV sentinel surveillance and the low-risk population as those not monitored by the HIV sentinel surveillance. (Money boys) MB are a subgroup of MSM who commercially sell sex to men. Male sexual transmission infections clinic attendees (male STI), long-distancing truck drivers (LDTD), and people who inject drugs (PWID) are all potential clients of female sex workers (FSW). All of them have a certain proportion that seeks commercial sexual contact with FSW. We defined "high-risk MSM" as those who satisfied at least one of the following [1]: (1) reported more than ten anal sex partners in the past six months; (2) reported condomless anal sex in the past six months; and (3) diagnosed with sexual transmission infections (STI) in the past six months. We defined the high-risk FSW as those who worked in high-tier entertainment venues [2], including hotels, nightclubs, star-ranked hotels with spas/saunas, karaoke clubs, dance halls, and pubs. Likewise, we defined the low-risk FSW as those who worked in low-tier entertainment venues [2], including small hairdressing salons, restaurants, temporary sublets, foot massages, unranked hostels, small hotels, and small pubs.

***Details for data collation***

Before data collation, we (LZ, MS, and SH) held the consensus workshop to confirm the criteria for all data sources. First, the recent HIV epidemic data reported by China CDC, UNAIDS, and WHO was prioritized to be used in our study. Second, the meta-analyses and system reviews that included data we needed were the secondary sources of data collation for us. Third, we used the mean value from several published articles if the data were not found in meta-analyses or national reports. The calculations and details for these means are shown in the next section, ‘Data estimation and calculation method.’ Fourth, some data were rare and only found in one published literature, so we collated the data from this unique literature. The literature we included fulfilled the following criteria: (1) provided a clear description of the demographic characteristics of the participants, such as age, marital status, education level, and recruitment location; (2) employed a probability sampling strategy; (3) reported inclusion and exclusion criteria for sample selection; (4) achieved an adequate response rate of more than 80%; and (5) conducted and reported appropriate statistical analyses.

After data collation, we (LZ, MS, and SH) presented these data to key stakeholders (DW, ZW) and collaborators from the National Center for AIDS/STD Control and Prevention, Chinese Center for Disease Control and Prevention, China. We discussed the data and their sources until a consensus was reached before the modeling exercise.

***Data estimation and calculation functions***

1. **Epidemiological and sexual behavioral data**

**1. Population size**

We employed distinct methodologies to estimate the population sizes across various groups. For MSM at high and low risk, population estimates were derived using the proportion of high-risk to low-risk MSM within the total MSM population. The 2021 population sizes for transgender women and MB were extrapolated based on the ratio of MSM to these groups. For high-risk and low-risk FSW, a similar methodology was applied, utilizing the ratio within the total FSW population. The population sizes for NMP and NFP were calculated based on the marriage rate among PLHIV and the gender ratio among PLHIV. LDTD population estimates were ascertained by averaging data from two published studies. For male STI, population size was determined by the prevalence of such infections and the number of corresponding cases. Lastly, the general male and female population aged 15-64, excluding high-risk groups, was calculated based on national demographic data. The specific values are detailed in Table S1.

**2. HIV prevalence**

For MB and transgender women, we used the mean value of several published studies. Other data were directly collated from published literature. The specific values are detailed in Table S2.

**3. HIV testing rate in the past year**

For general males and females, we estimated the HIV testing rate by using the total person-times one year in general populations divided by the total population size in China. Other data were directly collated from published literature. The specific values are detailed in Table S3.

**4. Antiretroviral therapy (ART) coverage rate in the past year**

Data were directly collated from published literature. The specific values are detailed in Table S4.

**5. Condom use over the last sex**

For MSM, the ratio of condom use of low-risk MSM to high-risk MSM was 3 to 4. According to the number of sexual partners and population size, we listed a formula *z*: (*n_h_***p_h_***r_h_*+*n_l_***p_l_***r_l_*)/(*n_h_***p_h_*+*n_l_***p_l_*)=*r_t_*, *r_h_*:*r_l_*=3:4.The detail as follows:

| parameter | The population size of high-risk MSM | The population size of low-risk MSM | The sexual partners of high-risk MSM | The sexual partners of low-risk MSM | The rate of MSM, high-risk MSM, and low-risk MSM condom use in the last sex, respectively |
| --- | --- | --- | --- | --- | --- |
|  | *n_h_* | *n_l_* | *p_h_* | *p_l_* | *r_t_*，*r_h_ ，r_l_* |
| value | 3.60 million | 8.40 million | 15 per year | 2.6 per year | *r_t_=*0.60 |

For transgender women, we assumed their characteristic of sexual behavior to be the same as the high-risk MSM, so we used the condom use rate of high-risk as the value of transgender women. For FSW and PWID, we calculated the mean value of several studies. For male STI, we calculated the mean value of condom use rate according to condom use rate in several kinds of sexual behavior at the last sex. Other data were directly collated from published literature. The specific values are detailed in Table S5.

**6. Willingness to test**

Data were directly collated from published literature. The specific values are detailed in Table S6.

**7. Frequency of sexual behaviors over the past year**

For FSW and MB, we used the frequency of commercial sex per month to multiply by twelve. Other data were directly collated from published literature. The specific values are detailed in Table S.

**8. The cost of linkage to care**

The cost of linkage to care is the cost of persuading one person who did not know his/her HIV status to seek HIV testing services through AIDS health promotion, such as knowledge pamphlets, counseling services, and other health education activities. One person who did not know his/her HIV status’ included those who did not know their HIV status ever and recent HIV status since engaging in a (high-) risk event. As the cost of linkage to care was only available for PWID ($8.1), we used PWID as a reference population and assumed the cost of linkage to care would be inversely proportional to the 'willingness to test' in the corresponding population. The cost of linkage to care of PWID was *C_PWID_*, the coverage of HIV testing among PWID was *R_PWID_*, the cost of linkage to care of other 13 populations was *C_p_*, and the willingness to test among other 13 populations was *R_p_*. The relationship between PWID and other populations was assumed to be satisfied by equation *Z*: *C_PWID_*R_PWID_=C_p_*R_p_*. We assumed the cost of linkage to care equal total advertising costs divided by the number of people persuaded and taken HIV test results from advertising intervention. We got the function of the cost of linkage to care among PWID:（5870000-2735110*2.1）/（9906-7739）= 126269/2167=58 yuan/person≈$8.12. According to the equation *Z*, we can calculate the costs of linkage to care of other populations (shown in **Table S8)**

**(2) The sexual behavior mixing matrix among 14 populations**

The sexual behavior mixing matrix delineates the patterns of sexual interactions among different risk subgroups. Within the context of our study's fourteen identified risk groups, we utilize a 14x14 matrix to represent the percentages of sexual activities occurring either within the same risk group or between different groups. In this matrix, the sum of all partnership percentages for each group equals 100%. Specifically, for a given population *i* (*i* =1,2,…, 14), the proportion of having sexual behavior with *j* (*j* =1,2,…, 14) population can be expressed as $p_{i,j}$. For example, as *i*=1,*j*=(1,2,...,14), $p_{1,1}+p_{1,2}+p_{1,3}+\ldots+p_{1,14}=1$_._ We calculated the proportion of having sexual behavior with the other 14 populations for each population by following two methods: (1) the direct data of components of sexual behavior over the past year for *i* population (e.g., the proportion of seeking commercial sex or heterosexual sex among MSM); (2) the ratio of the population size of *j* populations (*j* =1,2,…, 14) for the sexual partners over the past year of population *i*. The mixing matrix is shown in Figure S1.

**(3) The sexual condom use matrix among 14 populations**

We calculated the average rate of last condom use rate of sexual contact between two populations to estimate the sexual condom use matrix. For instance, the rate of condom use of high-risk MSM and low-risk MSM was 54.74% and 72.99%, respectively. The condom use rate of sexual acts between high-risk MSM and low-risk MSM was (54.74%+72.99%)/2=63.87%. The sexual condom use matrix is shown in Figure S2.

***Model calibration***

We assumed the transmission probability between male-to-male, male-to-female, and female-to-male was $\beta_{mm}$ , $\beta_{mf}$ and $\beta_{fm}$ respectively. We defined the upbound of $\beta_{mm}$ , $\beta_{mf}$ and $\beta_{fm}$ was 149, 16, and 8 per 10,000 person-year from published literature [3]. We used the nonlinear least-squares method (NLS) to estimate the transmission probability by fitting the annual HIV incidence rate. We divided the number of new HIV infections in each population ($I_{j}$) by per population size ($N_{j}$) to estimate the incidence rate of each population per year. We used the incidence rate without ART calculated by our model to fit with the annual incidence rate of each population from published literature (**Table S9**). As a result, $\beta_{mm}$, $\beta_{mf}$ and $\beta_{fm}$ was 75.6, 16.0 and 4.0 per 10,000 person-years by calibration. The total number of annual HIV infections calculated by our model was 144,795, similar to the 129,000 diagnosed number reported by the China CDC report.

***Marginal cost calculation and the simulation of the linkage to care cost***

1. **Calculation of linkage to care cost**

We assumed the per-capita cost of linkage to care for *i-th* population is constant ($c_{i}$) when the optimized testing rate ($x_{i}$) is below the willingness to test ($w_{i}$) (*i* = 1,2, ⋯, 14). As the optimized testing rate ($x_{i}$) increases beyond the willingness to test ($w_{i}$), the per-capita linkage to care cost would increase with the increment in each population's marginal testing rate. That is, with each additional 1% increase in the testing rate, more marginal costs would be required to link an individual to HIV testing and receive HIV care, which means that the increasing testing rate lowers the yield rate. We assumed that when the optimized testing rate $x_{i}$ exceeds the willingness to test $w_{i}$, each 1% increase in $x_{i}$ would result in a corresponding percentage increase $(q_{i})$ in $c_{i}$ ($q_{i}=1\%/(1-x_{i})$). Based on this assumption, the costs associated with linkage to care following a 1% increase in testing rate can be mathematically expressed as $(1+q_{i})c_{i}$.

1. **Simulation of the linkage to care cost**

We calculated the cost of linkage to care corresponding to each 1% increase in the testing rate over the willingness to test in each population. We presumed the relationship between testing rate and linkage to care cost followed an inversely proportional function. To fit the trend of the cost of linkage to care as the testing rate increases, we developed an inverse proportional function: $c_{i}'=q_{i}/(x_{i}+g_{i})+h_{i}$ .

where the $x_{i}$ presents optimized testing rate, $c_{i}'$ presents costs of linkage to care after optimization $q_{i}$, $g_{i}$, and $h_{i}$ are uncertain parameters. We fitted the cost of the linkage to care curve by using the nonlinear least-squares method (NLS), shown in Figure S4**.** The value of $q_{i}$, $g_{i}$, and $h_{i}$ were shown in Table S10.

***Result of sensitivity analysis without PMTCT***

To assess the robustness of our findings, we conducted a sensitivity analysis to evaluate the potential impact of removing the PMTCT program on the optimized allocation of resources for HIV testing. We evaluated the effect of removing the constraint of maintaining the latest reported maternal testing rate for pregnant women. We defined the low-bound and up-bound HIV testing rates for pregnant women as 0% and 100% (Figure S5-S7, Table S11-S12). Other data remain unchanged.

***Reference***

1. Zhang, L., et al., *Modelling the Epidemiological Impact and Cost-Effectiveness of PrEP for HIV Transmission in MSM in China.* AIDS Behav, 2019. **23**(2): p. 523-533.

2. Zhang, L., et al., *A systematic review and meta-analysis of the prevalence, trends, and geographical distribution of HIV among Chinese female sex workers (2000-2011): implications for preventing sexually transmitted HIV.* Int J Infect Dis, 2015. **39**: p. 76-86.

3. Patel, P., et al., *Estimating per-act HIV transmission risk: a systematic review.* Aids, 2014. **28**(10): p. 1509-19.

4. Xie, Z., et al., *The necessity of social support for transgender people in China.* Lancet, 2021. **397**(10269): p. 97.

5. Lu, F., et al., *Estimating the number of people at risk for and living with HIV in China in 2005: methods and results.* Sex Transm Infect, 2006. **82 Suppl 3**(Suppl 3): p. iii87-91.

6. Yang, Z., et al., *Estimating Changes in Population Size and Behavioral Characteristics in Men Who Have Sex With Men Between 2014 and 2019: Longitudinal Study.* JMIR Public Health Surveill, 2022. **8**(8): p. e34150.

7. He, N., et al., *HIV risks among two types of male migrants in Shanghai, China: money boys vs. general male migrants.* Aids, 2007. **21 Suppl 8**: p. S73-9.

8. Committee, C.N.N.C. *2019 China Drug Situation Report*. 2019.

9. Lau, M., *China’s sex industry flourishing despite dangerous conditions and corrupt police.* South China Morning Post, 2014. **3**.

10. Liang, F., et al., *[Analysis on the factors associated with HIV infection among female sex workers in Qinzhou, Guangxi, 2010-2018].* Applied Preventive Medicine., 2020. **26**(04): p. 279-283+286.

11. Chow, E.P., et al., *Risk behaviours among female sex workers in China: a systematic review and data synthesis.* PLoS One, 2015. **10**(3): p. e0120595.

12. Li, N., *[Epidemiological characteristics of HIV-infected and AIDS patients with different sexes of sexually transmitted men in Cixi City, 2009-2018].* Chinese Journal of Rural Medicine and Pharmacy, 2020. **27**(17): p. 63-64.

13. Chai, Y.P. and H.L. Wang, *[Epidemiologic characteristics of newly reported HIV infections and AIDS patients in Dongying city of China from 2010 to 2019].* Chinese Journal of Viral Diseases, 2021. **11**(06): p. 476-480.

14. Wu, Z., J.M. McGoogan, and R. Detels, *The Enigma of the Human Immunodeficiency Virus (HIV) Epidemic in China.* Clin Infect Dis, 2021. **72**(5): p. 876-881.

15. Lin, c.z., et al., *[Analysis on characteristics of AIDS patients receiving antiretroviral therapy, Fuzhou city, 2014-2018].* Preventive Medicine Tribune, 2021. **27**(09): p. 687-690.

16. Han, M.J., *[Analysis of the HIV/AIDS epidemic and prospects for prevention and treatment in China].* Chinese Journal of AIDS & STD, 2023. **29**(03): p. 247-250.

17. NCAIDS, NCSTD, and C. CDC, *[Update on the AIDS/STD epidemic in China the third quarter of 2018].* Chinese Journal of AIDS & STD, 2018. **24**(11): p. 1075.

18. Ye, X., J. Liu, and Z. Yi, *Trends in the Epidemiology of Sexually Transmitted Disease, Acquired Immune Deficiency Syndrome (AIDS), Gonorrhea, and Syphilis, in the 31 Provinces of Mainland China.* Med Sci Monit, 2019. **25**: p. 5657-5665.

19. Chen, W., N. Wei, and X. Huang, *[Risk factors for Neisseria gonorrhoeae and genital Chlamydia trachomatis infections among male outpatients attending sexually transmitted disease clinic in Zhuhai city, 2015-2018].* Practical Preventive Medicine., 2020. **27**(03): p. 296-299.

20. Du, X., Y. Tao, and S. Ge, *[Analysis on HIV/syphilis infection status and influencing factors of male STD outpatient patients, Zaozhuang city,2017-2019].* Preventive Medicine Tribune, 2021. **27**(03): p. 191-194.

21. Li, J. and M. Yun, *[China Logistics 40 years of four generations of truckers data report].* China Storage & Transport, 2019(01): p. 61-63.

22. Wen, X., *[Focus on the survival of the truck driver community].* Chinese Workers, 2018(08): p. 13.

23. Statistics, N.B.o. *China Statistical Yearbook 2021*. 2021; Available from: <http://www.stats.gov.cn/tjsj/ndsj/2021/indexch.htm>.

24. Jiang, J., *[Spatial distribution characteristics of maternal HIV infection in China in 2016].* National Medical Journal of China, 2018. **98**(41).

25. Yan, H., et al., *High HIV prevalence and associated risk factors among transgender women in China: a cross-sectional survey.* J Int AIDS Soc, 2019. **22**(11): p. e25417.

26. Shan, D., et al., *Correlates of HIV infection among transgender women in two Chinese cities.* Infect Dis Poverty, 2018. **7**(1): p. 123.

27. Xia, D., et al., *Psychosocial Problems and Condomless Anal Sex among Transgender Women in Two Cities of China: Study Based on the Syndemic Framework.* Int J Environ Res Public Health, 2022. **19**(23).

28. Yan, L., et al., *Awareness and Willingness to use HIV Pre-exposure Prophylaxis (PrEP) Among Trans Women in China: A Community-Based Survey.* AIDS Behav, 2021. **25**(3): p. 866-874.

29. Yu, M.H., et al., *Using latent class analysis to identify money boys at highest risk of HIV infection.* Public Health, 2019. **177**: p. 57-65.

30. Zhao, J., et al., *A comparison of HIV infection and related risk factors between money boys and noncommercial men who have sex with men in Shenzhen, China.* Sex Transm Dis, 2012. **39**(12): p. 942-8.

31. Chen, E.P., et al., *[HIV infection and associated factors of male sex workers among men who have sex with men].* Zhonghua Liu Xing Bing Xue Za Zhi, 2020. **41**(10): p. 1697-1702.

32. Huang, Y., et al., *Changes in Prevalence of HIV or Syphilis among Male Sex Workers and Non-Commercial Men Who Have Sex with Men in Shenzhen, China: Results of a Second Survey.* PLoS One, 2016. **11**(12): p. e0167619.

33. Cai, Y.M., et al., *[Factors associated with commercial sexual behavior among men who have sex with men in Shenzhen, China, in 2011-2015].* Zhonghua Yu Fang Yi Xue Za Zhi, 2016. **50**(11): p. 943-948.

34. Huang, Y., et al., *Correction: Changes in Prevalence of HIV or Syphilis among Male Sex Workers and Non-Commercial Men Who Have Sex with Men in Shenzhen, China: Results of a Second Survey.* PLoS One, 2017. **12**(3): p. e0175017.

35. Yan, H., et al., *Epidemiological and molecular characteristics of HIV infection among money boys and general men who have sex with men in Shanghai, China.* Infect Genet Evol, 2015. **31**: p. 135-41.

36. Wu, Z., et al., *History of the HIV Epidemic in China.* Curr HIV/AIDS Rep, 2019. **16**(6): p. 458-466.

37. Lu, H., et al., *HIV prevalence among drug users in China: a Meta-analysis.* Chinese Journal of AIDS & STD, 2021. **27**(04): p. 360-364.

38. He, N., *Research Progress in the Epidemiology of HIV/AIDS in China.* China CDC Wkly, 2021. **3**(48): p. 1022-1030.

39. Lu, M., et al., *[HIV positive rate from different detection methods in medical institutions in China: a Meta analysis].* Chinese Journal of Preventive Medicine, 2020. **54**(11): p. 1289-1294.

40. Zhang, X., et al., *Prevalence of HIV and syphilis infections among long-distance truck drivers in China: a data synthesis and meta-analysis.* Int J Infect Dis, 2013. **17**(1): p. e2-7.

41. Liu, X.J., J.M. McGoogan, and Z.Y. Wu, *Human immunodeficiency virus/acquired immunodeficiency syndrome prevalence, incidence, and mortality in China, 1990 to 2017: a secondary analysis of the Global Burden of Disease Study 2017 data.* Chin Med J (Engl), 2021. **134**(10): p. 1175-1180.

42. Best, J., et al., *Sexual behaviors and HIV/syphilis testing among transgender individuals in China: implications for expanding HIV testing services.* Sex Transm Dis, 2015. **42**(5): p. 281-5.

43. Wu, Z., et al., *HIV/AIDS in China: epidemiology, prevention and treatment*. 2019: Springer.

44. Hajarizadeh, B., et al., *Global, regional, and country-level coverage of testing and treatment for HIV and hepatitis C infection among people who inject drugs: a systematic review.* Lancet Glob Health, 2023. **11**(12): p. e1885-e1898.

45. Jiao, K., et al., *HIV testing frequency and associated factors among five key populations in ten cities of China: a cross-sectional study.* BMC Infect Dis, 2022. **22**(1): p. 195.

46. Cao, Y., *[Prevalence and Correlates of Syphilis and HIV Infection among Male Clients in STD Clinic,in ShandongProvince,from 2011 to 2015]*. 2018, Shandong University.

47. *The 8th National Academic Conference on HIV/AIDS*. 2023, National Center for AIDS/STD Control and Prevention.

48. Wang, X., et al., *Prevention of Mother-To-Child Transmission of HIV - China, 2011-2020.* China CDC Wkly, 2021. **3**(48): p. 1018-1021.

49. Reback, C.J. and D. Rünger, *Technology use to facilitate health care among young adult transgender women living with HIV.* AIDS Care, 2020. **32**(6): p. 785-792.

50. Zhang, W., *[Dynamic Model Prediction and Analysis of HIV/AIDS Epidemic among Men Who Have Sex with Men in China]*. 2017, Nanjing Medical University.

51. Bayer, A.M., et al., *The odyssey of linking to and staying in HIV care among male sex workers in Peru.* J hiv aids, 2017. **3**(1).

52. Ma, Y., et al., *The Human Immunodeficiency Virus Care Continuum in China: 1985-2015.* Clin Infect Dis, 2018. **66**(6): p. 833-839.

53. Dong, W., et al., *[HIV and syphilis infection and related medical treatment status of low-fee female sex workers in three provinces of China, 2012-2015].* Zhonghua Yu Fang Yi Xue Za Zhi, 2018. **52**(12): p. 1239-1242.

54. Na, H., *Emerging changes and characteristics of the HIV epidemic in China.* Shanghai Journal of Preventive Medicine, 2019. **31**(12): p. 963-967.

55. Zhou, H., et al., *Antiretroviral therapy among pregnant and postpartum women in China: A systematic review and meta-analysis.* Am J Infect Control, 2016. **44**(3): p. e25-35.

56. Li, J., et al., *A mathematical model of biomedical interventions for HIV prevention among men who have sex with men in China.* BMC Infect Dis, 2018. **18**(1): p. 600.

57. Zhu, Q., et al., *Analysis of drug sentinel sites information in Chuxiong County (2011-2016).* Journal of Dermatology and Venereology, 2019. **41**(03): p. 354-357.

58. Dong, S., et al., *[Study of high-risk behaviors and HIV and syphilis infection among drug users based on sentinel surveillance from 2015 to 2018].* Chinese Journal of AIDS & STD, 2020. **26**(05): p. 522-525.

59. Zhu, G., et al., *[Analysis of HIV infection status and influencing factors among drug users in Guigang City from 2009 to 2019].* The Journal of Practical Medicine, 2021. **37**(06): p. 806-810.

60. Zhang, X., et al., *[Results of AIDS sentinel surveillance among drug users during 2015-2019 in Fangshan district, Beijing].* Chinese Journal of AIDS & STD, 2021. **27**(04): p. 406-409.

61. Yuan, D., et al., *[Analysis on the Surveillance Results of AIDS Among Drug Users in Dazhu County 2013 to 2019].* Journal of Preventive Medicine Information, 2021. **37**(03): p. 340-344.

62. Liu, Y. and K. Shang, *[Survey on knowledge of AIDS and risk behaviors among drug users in Shizuishan City].* The Medical Forum, 2019. **23**(22): p. 3245-3246.

63. Zhao, P.Z., et al., *Uptake and correlates of chlamydia and gonorrhea testing among female sex workers in Southern China: a cross-sectional study.* BMC Public Health, 2021. **21**(1): p. 1477.

64. Ayele, W.M., et al., *Prevalence of Consistent Condom Use and Associated Factors among Serodiscordant Couples in Ethiopia, 2020: A Mixed-Method Study.* Biomed Res Int, 2021. **2021**: p. 9923012.

65. Ding, Z., *[The Study of High-risk Behavior and HIV Infection Status among Male Sexually Transmitted Disease Clinic Clients]*. 2017, Chinese Center for Disease Control and Prevention.

66. Xue, H., et al., *[Comparative analysis of high-risk sexual behavior of long-haul truck drivers in Yunnan border China, Laos and Myanmar].* Journal of Modern Medicine & Health, 2012. **28**(03): p. 474-475.

67. Kui, P., *[Epidemiologic Study of Genital Chlamydia Trachomatis Infection Based on A Community Population]*. 2019, Shandong Univerisity.

68. Jiang, Y., H. Yang, and J. Ren, *[Investigation and analysis of AIDS knowledge for female in Zhejiang].* International Journal of Epidemiology and Infectious Disease, 2009. **36**(2): p. 97-100.

69. Yu, W., *[Investigation of Knowledge, Attitude and Practice Related to AIDS and Factors of Influencing for Different People in the High Incidence of AIDS]*. 2012, Henan University.

70. Shrestha, R., et al., *Willingness to Use HIV Self-Testing and Associated Factors Among Transgender Women in Malaysia.* Transgend Health, 2020. **5**(3): p. 182-190.

71. Wang, L., et al., *[Analysis of the MSM’s Willingness to Detect HIV in Chaoyang District,Beijing].* Chinese Journal of AIDS & STD, 2015. **21**(11): p. 950-953.

72. Zhang, H., et al., *Analysis of high risk behavior of AIDS among self-employed and club-employed MSWs in Tianjin.* Chinese Journal of AIDS & STD, 2018. **24**(09): p. 908-911.

73. Guo, L., M. Han, and D. Yu, *[An analysis on the demand on HIV voluntary counseling and testing in*

*high risk population].* Disease Surveillance, 2006(07): p. 355-357.

74. Luo, X., et al., *[Survey on the Knowledge,Attitude,and Practice towards AIDS and Syphilis and the Prevalence of AIDS and Syphilis Infection Among Drug Users,Huangpu District,Guangzhou City,2012].* Preventive Medicine Tribune, 2014. **20**(10): p. 721-724.

75. Du, J., et al., *A mixed methods approach to identifying factors related to voluntary HIV testing among injection drug users in Shanghai, China.* Int J Infect Dis, 2012. **16**(7): p. e498-503.

76. Jingbin, P., *[The Study on the Utilization and Its Associated Factors of HIV VCT Clinic for Female Sex Workers in Jinan City, Shandong Province]*. 2010, Chinese Center for Disease Control and Prevention.

77. Shi, P., et al., *[The Effect of Enlarging HIV Testing Among STD Clinic Attendants in Wuhan].* Journal of Preventive Medicine Information, 2018. **34**(06): p. 733-736.

78. Hu, Q., et al., *[Analysis on the detection will of AIDS and syphilis in patients with sexually transmitted diseases and the influencing factors].* Preventive Medicine Tribune, 2017. **29**(06): p. 613-616.

79. Yun, F., et al., *[Survey on KAP towards STD/AIDS and willingness to VCT among Long-distance truck drivers in Nanfang Parking‚*

*Shanghai].* Chinese Journal of Health Education, 2007(12): p. 894-896.

80. Huo, J., et al., *[Willingness for HIV testing and associated factors among people for health examination in Kunming and Zhaotong two prefectures].* Journal of Dermatology and Venereology, 2019. **41**(05): p. 635-638.

81. Liu, B., et al., *What factors hinder ethnic minority women in rural China from getting antenatal care? A retrospective data analysis.* BMJ Open, 2019. **9**(8): p. e023699.

82. Durex. *Durex Global Welfare Index*. 2011; Available from: <https://baike.baidu.com/item/%E6%9D%9C%E8%95%BE%E6%96%AF%E5%85%A8%E7%90%83%E6%80%A7%E7%A6%8F%E6%8C%87%E6%95%B0/1151845#reference-1-2863354-wrap>.

83. Yang, G., et al., *Analyzing cost-effectiveness on prevention and therapy of AIDS by cleaning needle/syringe exchange programs in Yunnan Province.* Medicine and Pharmacy of Yunnan, 2021. **42**(03): p. 212-216+227.

84. Zhang, G., X. Wang, and J. Li, *Prevalence of hepatitis C virus infection and risk factors among drug users in Shijingshan district,Beijing.* Disease Surveillance, 2009. **24**(11): p. 872-874.

85. Shan, D., et al., *HIV incidence and risk factors among transgender women and cisgender men who have sex with men in two cities of China: a prospective cohort study.* Infect Dis Poverty, 2022. **11**(1): p. 26.

86. You, X., et al., *HIV incidence and sexual behavioral correlates among 4578 men who have sex with men (MSM) in Chengdu, China: a retrospective cohort study.* BMC Public Health, 2021. **21**(1): p. 802.

87. Yang, L., et al., *The changing trends of HIV-1 prevalence and incidence from sentinel surveillance of five sub-populations in Yunnan, China, 2001-2010.* BMC Public Health, 2015. **15**: p. 376.

88. Su, Y., et al., *Loss to follow-up and HIV incidence in female sex workers in Kaiyuan, Yunnan Province China: a nine year longitudinal study.* BMC Infect Dis, 2016. **16**(1): p. 526.

89. Ma, Y., et al., *Long-Term Changes of HIV/AIDS Incidence Rate in China and the U.S. Population From 1994 to 2019: A Join-Point and Age-Period-Cohort Analysis.* Front Public Health, 2021. **9**: p. 652868.

**Figure S1 The distribution of sexual behaviors in each population group.**

TW=transgender women, HRMSM=high-risk men who have sex with men, MB=money boys, LRMSM=low-risk men who have sex with men, PWID=people who inject drugs, HRFSW=high-risk female sex workers, LRFSW=low-risk female sex workers, NMP=HIV-negative male partners of serodiscordant couples, NFP=HIV-negative female partners of serodiscordant couples, Male STI=male sexual transmission infections clinic attendees, GM=general males, GF=general females, PW=pregnant women, LDTD=long-distancing truck drivers. Each row represents the proportion of the frequency of sexual behaviors in each population and adds up to 1. 0 means no sexual contact occurs between these two populations.


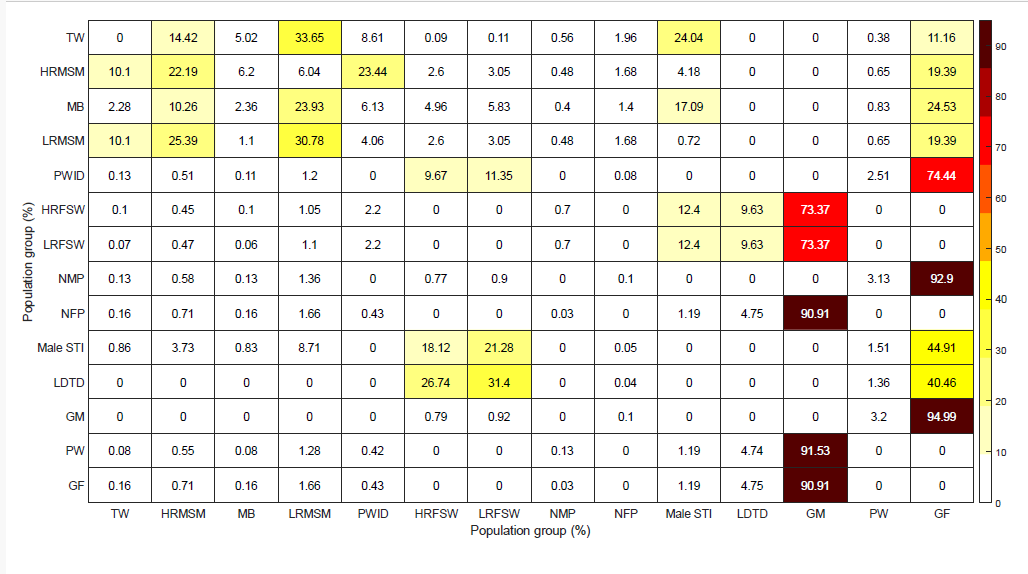


**Figure S2 The distribution of condom use rate in each population group.**

TW=transgender women, HRMSM=high-risk men who have sex with men, MB=money boys, LRMSM=low-risk men who have sex with men, PWID=people who inject drugs, HRFSW=high-risk female sex workers, LRFSW=low-risk female sex workers, NMP=HIV-negative male partners of serodiscordant couples, NFP=HIV-negative female partners of serodiscordant couples, Male STI=male sexual transmission infections clinic attendees, GM=general males, GF=general females, PW=pregnant women, LDTD=long-distancing truck drivers. Each value represents the rate of condom use of each sexual contact between two population groups. 0 means that no sexual contact occurs between these two populations.


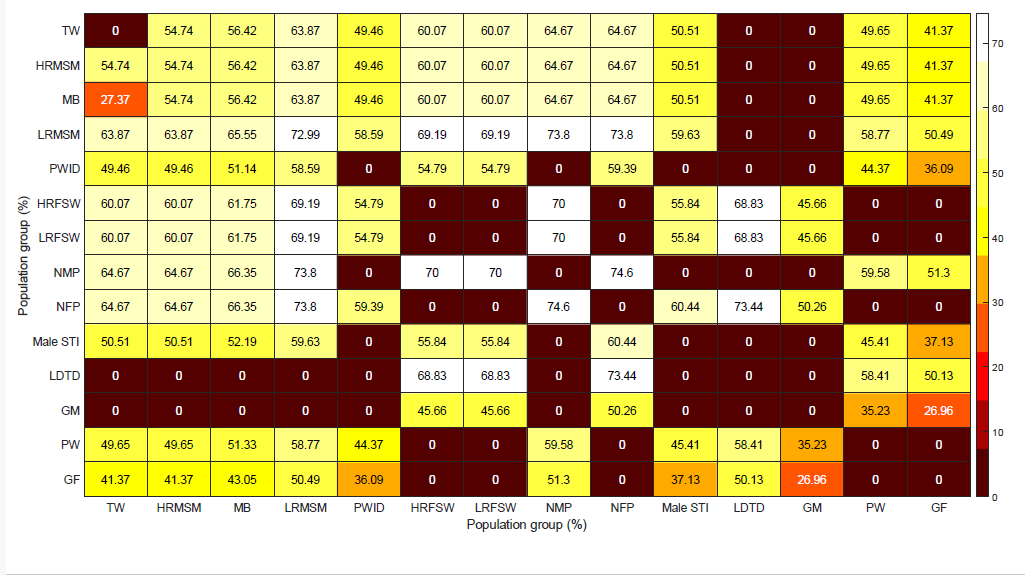


**Figure S3. Model calibration and data fitting based on HIV incidence.**

TW=transgender women, HRMSM=high-risk men who have sex with men, MB=money boys, LRMSM=low-risk men who have sex with men, PWID=people who inject drugs, HRFSW=high-risk female sex workers, LRFSW=low-risk female sex workers, SC=sero-discordant couples, Male STI=male sexual transmission infections clinic attendees, GM=general males, GF=general females, PW=pregnant women, LDTD=long-distancing truck drivers.

(a) the incidence rate without ART (the red line represents incidence from current data, and the black line represents incidence by model). (b) the incidence rate calculated by model with ART


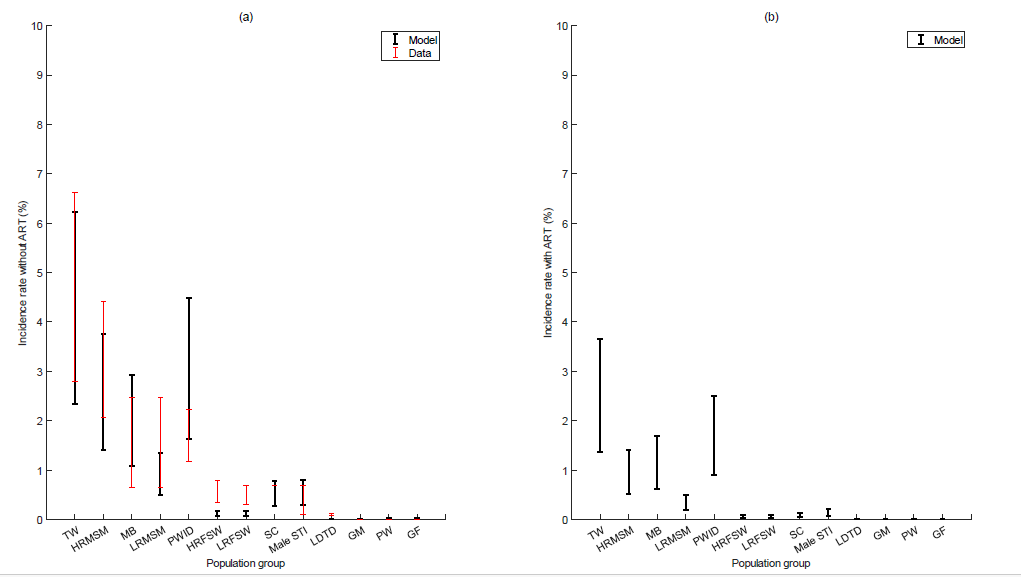


**Figure S4. The relationship between the testing rate and the cost of link-to-test among each population.**

TW=transgender women, HRMSM=high-risk men who have sex with men, MB=money boys, LRMSM=low-risk men who have sex with men, PWID=people who inject drugs, HRFSW=high-risk female sex workers, LRFSW=low-risk female sex workers, NMP=HIV-negative male partners of serodiscordant couples, NFP=HIV-negative female partners of serodiscordant couples, Male STI=male sexual transmission infections clinic attendees, GM=general males, GF=general females, PW=pregnant women, LDTD=long-distancing truck drivers. The blue line represents the fitting curve, and the red spot represents the cost of link-to-test at various optimized testing rates.


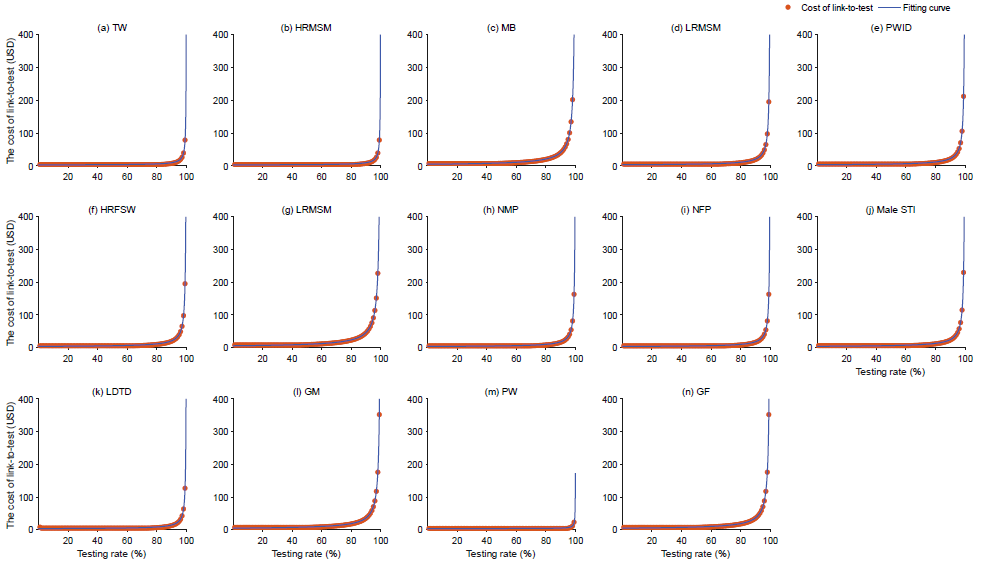


**Figure S5 HIV testing coverage in 14 Chinese populations at baseline and five scenarios of resource allocations without PMTCT.**

TW=transgender women, HRMSM=high-risk men who have sex with men, MB=money boys, LRMSM=low-risk men who have sex with men, PWID=people who inject drugs, HRFSW=high-risk female sex workers, LRFSW=low-risk female sex workers, NMP=HIV-negative male partners of serodiscordant couples, NFP=HIV-negative female partners of serodiscordant couples, Male STI=male sexual transmission infections clinic attendees, GM=general males, GF=general females, PW=pregnant women, LDTD=long-distancing truck drivers. Overall represents the proportion of detecting among total annual new infections.


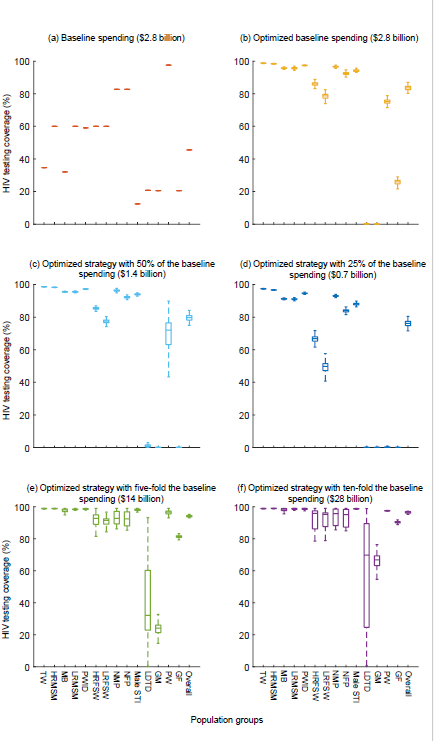


**Figure S6 The cost of detecting one HIV infection case under five scenarios of different total costs without PMTCT.**

TW=transgender women, HRMSM=high-risk men who have sex with men, MB=money boys, LRMSM=low-risk men who have sex with men, PWID=people who inject drugs, HRFSW=high-risk female sex workers, LRFSW=low-risk female sex workers, NMP=HIV-negative male partners of serodiscordant couples, NFP=HIV-negative female partners of serodiscordant couples, Male STI=male sexual transmission infections clinic attendees, GM=general males, GF=general females, PW=pregnant women, LDTD=long-distancing truck drivers. Overall represents the cost of detecting one infection case among whole population groups.


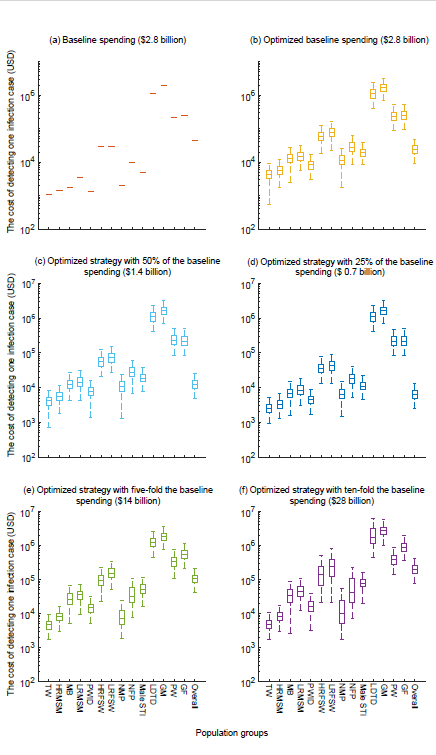


**Figure S7 The proportion of spending for HIV testing among 14 populations under baseline and five scenarios of different total costs without PMTCT**

The proportion of spending < 1% was not labeled in the plot.

TW=transgender women, HRMSM=high-risk men who have sex with men, MB=money boys, LRMSM=low-risk men who have sex with men, PWID=people who inject drugs, HRFSW=high-risk female sex workers, LRFSW=low-risk female sex workers, NMP=HIV-negative male partners of serodiscordant couples, NFP=HIV-negative female partners of serodiscordant couples, Male STI=male sexual transmission infections clinic attendees, GM=general males, GF=general females, PW=pregnant women, LDTD=long-distancing truck drivers. Overall represents the cost of detecting one infection case among whole population groups.


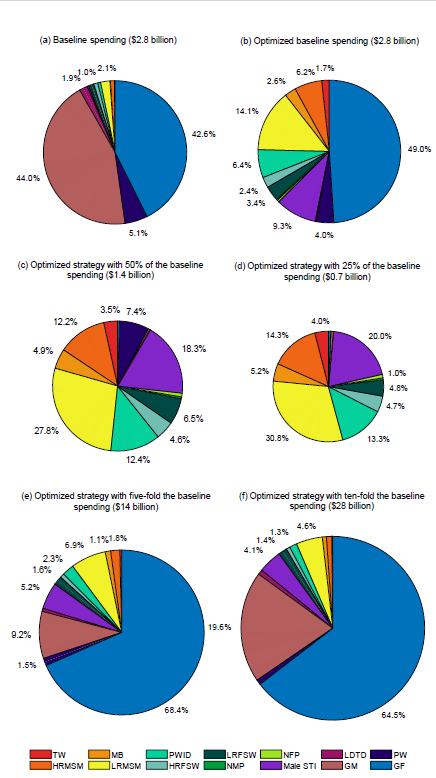


**Table S1 Data source of the latest reported population size**

| **Population** | **Latest reported population size (millions)** | **Calculation function** | **The latest reported years of data** | **Reference** |
| --- | --- | --- | --- | --- |
| Transgender women | 0.83 | 12*0.4/(5.35+0.4) | 2021 | [4, 5] |
| High-risk MSM | 3.6 | 12*0.3 | 2021 | [1, 6] |
| Money boys | 0.80 | 12*0.38/(5.35+0.38) | 2021 | [5, 7] |
| Low-risk MSM | 8.4 | 12*0.7 | 2021 | [1, 6] |
| PWID | 2.15 | direct data | 2019 | [8] |
| High-risk FSW | 3.68 | (2.76+4.6)/2 | 2015 | [2, 9-11] |
| Low-risk FSW | 4.32 | (3.24+5.4)/2 | 2015 | [2, 9-11] |
| NMP | 0.14 | 1.223*0.5094*3.6/(1+3.6) | 2020 | [12-16] |
| NFP | 0.49 | 1.223*0.5094*1/(1+3.6) | 2020 | [12-16] |
| Male STI | 6 | (7.11*3.2/4.2+5.38*5.2/6.2+42.91*1/2)*14/0.1 | 2020 | [17-20] |
| LDTD | 24 | (18+30)/2 | 2019 | [21, 22] |
| General males | 459.62 | 505.54-3.60-8.40-0.80-0.83-2.15-0.14-6.0-24.0 | 2021 | [23] |
| Pregnant women | 15 | direct data | 2018 | [24] |
| General females | 445.15 | 468.64-3.68-4.32-0.488-15 | 2021 | [23] |

MSM: men who have sex with men; PWID: people who inject drugs; FSW: female sex workers; NMP: HIV-negative male of serodiscordant couples; NFP: HIV-negative female of serodiscordant couples; Male STI: male sexual transmission infections clinic attendees;

LDTD: long-distancing truck drivers;

**Table S2 Data source of the HIV prevalence**

| **Population** | **HIV prevalence (%)** | **Calculation function** | **The latest reported years of data** | **Reference** |
| --- | --- | --- | --- | --- |
| Transgender women | 12.1 | (37+38+28+48)/(250+498+250+247) | 2018-2022^#^ | [25-28] |
| High-risk MSM | 10.7 | direct data | 2019 | [1] |
| Money boys | 7.8 | (38+38+27+14+297+14+57)/(535+489+3040+489+500+850+330) | 2012-2019^#^ | [29-35] |
| Low-risk MSM | 5.3 | direct data | 2019 | [1, 36] |
| PWID | 2.0 | direct data | 2021 | [37] |
| High-risk FSW | 1.1 | direct data | 2015 | [2] |
| Low-risk FSW | 0.74 | direct data | 2015 | [2] |
| NMP | 0.34 | direct data | 2021 | [38] |
| NFP | 0.34 | direct data | 2021 | [38] |
| Male STI | 0.3 | direct data | 2020 | [39] |
| LDTD | 0.19 | direct data | 2013 | [40] |
| General males | 0.065 | direct data | 2021 | [41] |
| Pregnant women | 0.034 | direct data | 2018 | [24] |
| General females | 0.024 | direct data | 2021 | [41] |

^#^denoted the reported year of the data source used to calculate the mean value.

MSM: men who have sex with men; PWID: people who inject drugs; FSW: female sex workers; NMP: HIV-negative male of serodiscordant couples; NFP: HIV-negative female of serodiscordant couples; Male STI: male sexual transmission infections clinic attendees;

LDTD: long-distancing truck drivers;

**Table S3 Data source of HIV testing rate over the past 12 months**

| **Population** | **HIV testing rate over the past 12 months (%)** | **Calculation function** | **The latest reported years of data** | **Reference** |
| --- | --- | --- | --- | --- |
| Transgender women | 34.6 | direct data | 2015 | [42] |
| High-risk MSM | 60 | direct data | 2021 | [16] |
| Money boys | 32 | direct data | 2019 | [43] |
| Low-risk MSM | 60 | direct data | 2021 | [16] |
| PWID | 59 | direct data | 2021 | [44] |
| High-risk FSW | 60 | direct data | 2021 | [16] |
| Low-risk FSW | 60 | direct data | 2021 | [16] |
| NMP | 82.7 | direct data | 2021 | [45] |
| NFP | 82.7 | direct data | 2021 | [45] |
| Male STI | 12.5 | direct data | 2018 | [46] |
| LDTD | 20.7 | direct data | 2021 | [14] |
| General males | 20.6 | [322.8-(0.83*0.346+3.60*0.6+0.80*0.32+8.40*0.6+2.15*0.59+8*0.6+0.63*0.827+6*0.125+24*0.207+15*0.975)]/1400 | 2021 | [23, 47] |
| Pregnant women | 97.5 | direct data | 2021 | [48] |
| General females | 20.6 | [322.8-(0.83*0.346+3.60*0.6+0.80*0.32+8.40*0.6+2.15*0.59+8*0.6+0.63*0.827+6*0.125+24*0.207+15*0.975)]/1400 | 2021 | [23, 47] |

MSM: men who have sex with men; PWID: people who inject drugs; FSW: female sex workers; NMP: HIV-negative male of serodiscordant couples; NFP: HIV-negative female of serodiscordant couples; Male STI: male sexual transmission infections clinic attendees;

LDTD: long-distancing truck drivers;

**Table S4 Data source of ART coverage rate over the past 12 months**

| **Population** | **ART coverage rate over the past 12 months (%)** | **The latest reported years of data** | **Reference** |
| --- | --- | --- | --- |
| Transgender women | 48.5 | 2020 | [49] |
| High-risk MSM | 73.5 | 2017 | [50] |
| Money boys | 50.0 | 2017 | [51] |
| Low-risk MSM | 73.5 | 2017 | [50] |
| PWID | 52.0 | 2018 | [52] |
| High-risk FSW | 55.4 | 2018 | [53] |
| Low-risk FSW | 55.4 | 2018 | [53] |
| NMP | 96.6 | 2021 | [38] |
| NFP | 96.6 | 2021 | [38] |
| Male STI | 86.0 | 2019 | [54] |
| LDTD | 86.6 | 2019 | [54] |
| General males | 86.6 | 2019 | [54] |
| Pregnant women | 95.0 | 2016 | [55] |
| General females | 86.6 | 2019 | [54] |

MSM: men who have sex with men; PWID: people who inject drugs; FSW: female sex workers; NMP: HIV-negative male of serodiscordant couples; NFP: HIV-negative female of serodiscordant couples; Male STI: male sexual transmission infections clinic attendees;

LDTD: long-distancing truck drivers;

**Table S5 Data source of condom use rate over the last sex**

| Population | Condom use rate over the last sex (%) | Calculation function | The latest reported years of data | Reference |
| --- | --- | --- | --- | --- |
| Transgender women | 54.7 | see formula *z* | 2021 | [1, 6, 16, 56] |
| High-risk MSM | 54.7 | see formula *z* | 2021 | [1, 6, 16, 56] |
| Money boys | 58.1 | direct data | 2019 | [29] |
| Low-risk MSM | 72.9 | see formula *z* | 2021 | [1, 6, 16, 56] |
| PWID | 44.2 | (540+646+1578+176+279+279)/ (1587+1584+3345+341+955+676) | 2020 | [57-62] |
| High-risk FSW | 65.4 | (304+167+1537+125+169)/(354+193+1631+134+204) | 2021 | [63] |
| Low-risk FSW | 65.4 | (304+167+1537+125+169)/(354+193+1631+134+204) | 2021 | [63] |
| NMP | 74.6 | direct data | 2021 | [64] |
| NFP | 74.6 | direct data | 2021 | [64] |
| Male STI | 46.3 | (404*0.653+218*0.422+140*0.5+59*0.422+26*0.422)/848 | 2017 | [65] |
| LDTD | 72.3 | direct data | 2012 | [66] |
| General males | 25.9 | direct data | 2019 | [67] |
| Pregnant women | 44.6 | direct data | 2009 | [68] |
| General females | 28.0 | direct data | 2019 | [69] |

MSM: men who have sex with men; PWID: people who inject drugs; FSW: female sex workers; NMP: HIV-negative male of serodiscordant couples; NFP: HIV-negative female of serodiscordant couples; Male STI: male sexual transmission infections clinic attendees;

LDTD: long-distancing truck drivers;

**Table S6 Data source of willingness to test**

| **Population** | **Willingness to test (%)** | **The latest reported years of data** | **Reference** |
| --- | --- | --- | --- |
| Transgender women | 47.60 | 2020 | [70] |
| High-risk MSM | 65.00 | 2015 | [71] |
| Money boys | 63.30 | 2018 | [72] |
| Low-risk MSM | 65.00 | 2015 | [71] |
| PWID | 44.70 | 2014 | [73-75] |
| High-risk FSW | 69.00 | 2010 | [76] |
| Low-risk FSW | 69.00 | 2010 | [76] |
| NMP | 82.70 | 2021 | estimated |
| NFP | 82.70 | 2021 | estimated |
| Male STI | 61.60 | 2018 | [77, 78] |
| LDTD | 80.10 | 2007 | [79] |
| General males | 51.50 | 2019 | [80] |
| Pregnant women | 94.60 | 2019 | [81] |
| General females | 51.50 | 2019 | [80] |

MSM: men who have sex with men; PWID: people who inject drugs; FSW: female sex workers; NMP: HIV-negative male of serodiscordant couples; NFP: HIV-negative female of serodiscordant couples; Male STI: male sexual transmission infections clinic attendees;

LDTD: long-distancing truck drivers;

**Table S7 Data source of frequency of sexual behaviors over the past year**

| **Population** | **Frequency of sexual behaviors** | **Calculation function** | **The latest reported years of data** | **Reference** |
| --- | --- | --- | --- | --- |
| Transgender women | 122 | direct data | 2011 | [82] |
| High-risk MSM | 122 | direct data | 2011 | [82] |
| Money boys | 168 | 14*12 | 2019 | [29] |
| Low-risk MSM | 122 | direct data | 2011 | [82] |
| PWID | 122 | direct data | 2011 | [82] |
| High-risk FSW | 336 | 28*12 | 2021 | [63] |
| Low-risk FSW | 336 | 28*12 | 2021 | [63] |
| NMP | 122 | direct data | 2011 | [82] |
| NFP | 122 | direct data | 2011 | [82] |
| Male STI | 122 | direct data | 2011 | [82] |
| LDTD | 122 | direct data | 2011 | [82] |
| General males | 122 | direct data | 2011 | [82] |
| Pregnant women | 122 | direct data | 2011 | [82] |
| General females | 122 | direct data | 2011 | [82] |

MSM: men who have sex with men; PWID: people who inject drugs; FSW: female sex workers; NMP: HIV-negative male of serodiscordant couples; NFP: HIV-negative female of serodiscordant couples; Male STI: male sexual transmission infections clinic attendees;

LDTD: long-distancing truck drivers;

**Table S8 Data source of the cost of linkage to care**

| **Population** | **The cost of linkage to care (USD)** | **The latest reported years of data** | **Reference** |
| --- | --- | --- | --- |
| Transgender women | 7.6 | 2016 | estimated |
| High-risk MSM | 5.58 | 2016 | estimated |
| Money boys | 5.73 | 2016 | estimated |
| Low-risk MSM | 5.58 | 2016 | estimated |
| PWID | 8.12 | 2016 | [57, 83, 84] |
| High-risk FSW | 5.26 | 2016 | estimated |
| Low-risk FSW | 5.26 | 2016 | estimated |
| NMP | 4.03 | 2016 | estimated |
| NFP | 4.03 | 2016 | estimated |
| Male STI | 5.89 | 2016 | estimated |
| LDTD | 4.53 | 2016 | estimated |
| General males | 7.05 | 2016 | estimated |
| Pregnant women | 3.84 | 2016 | estimated |
| General females | 7.05 | 2016 | estimated |

MSM: men who have sex with men; PWID: people who inject drugs; FSW: female sex workers; NMP: HIV-negative male of serodiscordant couples; NFP: HIV-negative female of serodiscordant couples; Male STI: male sexual transmission infections clinic attendees;

LDTD: long-distancing truck drivers;

**Table S9 HIV incidence in each population for model calibration**

| **Population** | **HIV incidence (%; 95%CI)** | **Reference** |
| --- | --- | --- |
| Transgender women | 4.42 (2.80-6.62) | [85] |
| High-risk MSM | 3.09 (2.07-4.41) | [86] |
| Money boys | 1.35 (0.65-2.48) | [85] |
| Low-risk MSM | 1.35 (0.65-2.48) | [85] |
| PWID | 1.7 (1.17-2.24) | [87] |
| High-risk FSW | 0.57 (0.35-0.79) | [88] |
| Low-risk FSW | 1.35 (0.65-2.48) | [88] |
| Serodiscordant couples | 0.34 | [38] |
| Male STI | 0.40 (0.11-0.69) | [87] |
| LDTD | 0.0964 (0.0730-0.0120) | [89] |
| General males | 0.0033 (0.0025-0.0041) | [89] |
| Pregnant women | 0.0015 (0.0011-0.0019) | [89] |
| General females | 0.0015 (0.0011-0.0019) | [89] |

MSM: men who have sex with men; FSW: female sex workers; Male STI: male sexual transmission infections clinic attendees; LDTD: long-distancing truck drivers;

PWID: people who inject drugs.

**Table S10 The value of three parameters by data fitting.**

| **Population** | $\boldsymbol{q}_{\boldsymbol{i}}$ | $\boldsymbol{g}_{\boldsymbol{i}}$ | $\boldsymbol{h}_{\boldsymbol{i}}$ |
| --- | --- | --- | --- |
| Transgender women | -0.7 | -1.0 | 2.5 |
| High-risk MSM | -0.7 | -1.0 | 2.5 |
| Money boys | -4.0 | -1.0 | 1.2 |
| Low-risk MSM | -1.9 | -1.0 | 1.8 |
| PWID | -2.1 | -1.0 | 1.7 |
| High-risk FSW | -1.9 | -1.0 | 1.8 |
| Low-risk FSW | -4.5 | -1.0 | 1.1 |
| NMP | -1.6 | -1.0 | 2.0 |
| NFP | -1.6 | -1.0 | 2.0 |
| Male STI | -2.3 | -1.0 | 1.6 |
| LDTD | -1.2 | -1.0 | 2.2 |
| General males | -3.5 | -1.0 | 1.2 |
| Pregnant women | -0.2 | -1.0 | 3.3 |
| General females | -3.5 | -1.0 | 1.2 |

MSM: men who have sex with men; PWID: people who inject drugs; FSW: female sex workers; NMP: HIV-negative male of serodiscordant couples; NFP: HIV-negative female of serodiscordant couples; Male STI: male sexual transmission infections clinic attendees;

LDTD: long-distancing truck drivers.

**Table** **S11 The cost of detecting one** **infection case and diagnoses number for each population in baseline and optimized HIV testing strategy without PMTCT.**

| **Population** | **Estimated new infections in 12 months** | **Incidence rate (per 10 thousand)** | **Baseline testing strategy** | | | | **Optimized testing strategy** | | | |
| --- | --- | --- | --- | --- | --- | --- | --- | --- | --- | --- |
|  |  |  | **Baseline testing rate (%)** | **Diagnoses of new infection** | **Cost of detecting one HIV case (USD)** | **Allocation of HIV resources (%)** | **Optimized testing rate(%)**  **(median,**  **IQR)** | **Diagnoses of new infection**  **(median,**  **IQR)** | **Cost of detecting one HIV case (USD)**  **(median,**  **IQR)** | **Allocation of HIV resources (%)** |
| Transgender women | 11743  (8968-15451) | 141.5  (108.1-186.2) | 34.6 | 4063  (3103-5346) | 996  (757-1305) | 0.1 | 98.7  (98.7-98.8) | 11592  (8848-15260) | 4228  (3215-5463) | 1.7  (1.7-1.8) |
| High-risk MSM | 31962  (24289-42228) | 88.8  (67.5-117.3) | 60.0 | 19177  (14573-25337) | 1356  (1026-1784) | 0.9 | 98.4  (98.3-98.4) | 31440  (23874-41568) | 5578  (4264-7247) | 6.2  (6.0-6.4) |
| Money boys | 5798  (4437-7568) | 72.5  (55.5-94.6) | 32.0 | 1855  (1420-2422) | 1671  (1280-2183) | 0.1 | 95.7  (95.5-95.9) | 5548  (4237-7256) | 13188  (9900-17124) | 2.6  (2.4-2.7) |
| Low-risk MSM | 28422  (21098-38175) | 33.8  (25.1-45.4) | 60.0 | 17053  (12659-22905) | 3483  (2593-4692) | 2.1 | 95.6  (95.3-95.9) | 27185  (20096-36628) | 14622  (11189-19290) | 14.1  (13.1-15.1) |
| PWID | 22652  (17403-29588) | 105.5  (81.0-137.7) | 59.0 | 13365  (10268-17457) | 1391  (1065-1810) | 0.7 | 97.3  (97.3-97.4) | 22049  (16928-28830) | 8227  (6151-10540) | 6.4  (6.2-6.5) |
| High-risk FSW | 1379  (1027-1849) | 3.7  (2.8-5.0) | 60.0 | 827  (616-1109) | 30246  (22561-40604) | 0.9 | 86.1  (85.2-86.7) | 1187  (875-1602) | 56733  (42828-75996) | 2.4  (2.3-2.5) |
| Low-risk FSW | 1624  (1209-2177) | 3.8  (2.8-5.0) | 60.0 | 974  (726-1306) | 30153  (22489-40490) | 1.0 | 78.7  (77.3-79.6) | 1278  (935-1733) | 75423  (56525-98077) | 3.4  (3.2-3.6) |
| NMP | 676  (501-908) | 48.3  (35.8-64.8) | 82.7 | 559  (415-751) | 2216  (1650-2989) | 0.0 | 96.6  (96.3-96.8) | 653  (483-879) | 11302  (8315-15449) | 0.3  (0.2-0.3) |
| NFP | 500  (370-672) | 10.2  (7.6-13.7) | 82.7 | 414  (306-556) | 10292  (7655-13902) | 0.2 | 92.5  (91.8-92.9) | 463  (340-625) | 27957  (21206-37606) | 0.5  (0.4-0.5) |
| Male STI | 14487  (11564-18524) | 24.1  (19.3-30.9) | 12.5 | 1811  (1446-2315) | 5000  (3911-6264) | 0.3 | 94.1  (93.8-94.6) | 13629  (10845-17518) | 19337  (14974-24421) | 9.3  (8.8-9.9) |
| LDTD | 212  (159-278) | 0.1  (0.1-0.1) | 20.7 | 44  (33-57) | 1198893  (915114-1600556) | 1.9 | 0.2  (0.1-0.3) | 0  (0-1) | 1086240  (823059-1447116) | 0.0  (0.0-0.0) |
| General males | 3004  (2380-3944) | 0.1  (0.1-0.1) | 20.6 | 619  (491-813) | 2005013  (1527140-2530224) | 44.0 | 0.1  (0.1-0.1) | 3  (2-4) | 1644273  (1271244-2059614) | 0.2  (0.2-0.2) |
| Pregnant women | 649  (481-888) | 0.4  (0.3-0.6) | 97.5 | 633  (469-866) | 228746  (167103-308817) | 5.1 | 75.1  (74.2-76.1) | 488  (357-676) | 231779  (172770-311932) | 4.0  (3.9-4.1) |
| General females | 21687  (16268-29587) | 0.5  (0.4-0.7) | 20.6 | 4472  (3354-6101) | 269046  (197208-358671) | 42.6 | 25.9  (24.8-26.9) | 5614  (4029-7965) | 244026  (182384-326944) | 49.0  (46.5-51.1) |
| Overall | 144795  (110154-191837) | 1.5  (1.1-2.0) | 45.5* | 65867  (49877-87342) | 42852  (32316-56590) | 100 | 83.6*  (82.5-84.6) | 121083  (90893-162219) | 23406  (18141-30399) | 100 |

IQR: interquartile range. * represents the timely diagnosis rate among total annual new infections.

MSM: men who have sex with men; PWID: people who inject drugs; FSW: female sex workers; NMP: HIV-negative male of serodiscordant couples; NFP: HIV-negative female of serodiscordant couples; Male STI: male sexual transmission infections clinic attendees; LDTD: long-distancing truck drivers.

**Table** **S12 The cost of detecting one** **infection case and diagnoses number for each population at four different scenarios of resource allocations without PMTCT**

| **Population** | **Optimized strategy with 50% of the baseline spending** | | | | **Optimized strategy with 25% of the baseline spending** | | | |
| --- | --- | --- | --- | --- | --- | --- | --- | --- |
|  | **Optimized testing rate (%)**  **(median,**  **IQR)** | **Diagnoses of new infection**  **(median,**  **IQR)** | **Cost of detecting one HIV case (USD)**  **(median,**  **IQR)** | **Allocation of HIV resources (%)** | **Optimized testing rate (%)**  **(median,**  **IQR)** | **Diagnoses of new infection**  **(median,**  **IQR)** | **Cost of detecting one HIV case (USD)**  **(median,**  **IQR)** | **Allocation of HIV resources (%)** |
| Transgender women | 98.7  (98.6-98.7) | 11587  (8846-15250) | 4154  (3156-5369) | 3.5  (3.3-3.5) | 97.4  (97.3-97.5) | 11437  (8729-15059) | 2479  (1896-3255) | 4.0  (3.9-4.0) |
| High-risk MSM | 98.3  (98.3-98.3) | 31423  (23868-41525) | 5412  (4166-7064) | 12.2  (11.8-12.3) | 96.7  (96.6-96.7) | 30896  (23468-40839) | 3275  (2542-4252) | 14.3  (14.1-14.5) |
| Money boys | 95.5  (95.4-95.6) | 5540  (4234-7237) | 12404  (9503-16419) | 4.9  (4.8-5.0) | 91.2  (91.1-91.4) | 5288  (4041-6914) | 6913  (5331-9033) | 5.2  (5.1-5.3) |
| Low-risk MSM | 95.5  (95.3-95.7) | 27154  (20113-36526) | 14190  (10852-19157) | 27.8  (26.6-28.6) | 91.1  (90.8-91.3) | 25888  (19152-34843) | 8456  (6371-11309) | 30.8  (30.0-31.5) |
| PWID | 97.2  (97.2-97.3) | 22029  (16913-28791) | 7785  (6000-10184) | 12.4  (12.0-12.5) | 94.6  (94.4-94.8) | 21432  (16436-28050) | 4436  (3408-5677) | 13.3  (13.0-13.7) |
| High-risk FSW | 85.4  (84.9-85.8) | 1178  (872-1586) | 55184  (41492-73599) | 4.6  (4.5-4.8) | 66.9  (65.3-67.9) | 922  (671-1256) | 35998  (27221-47699) | 4.7  (4.5-4.9) |
| Low-risk FSW | 77.6  (76.6-78.2) | 1260  (927-1703) | 72179  (54261-95336) | 6.5  (6.2-6.7) | 49.8  (47.2-51.5) | 810  (571-1121) | 41913  (31636-55167) | 4.8  (4.5-5.2) |
| NMP | 96.4  (96.0-96.6) | 652  (481-877) | 10780  (7955-14629) | 0.5  (0.4-0.5) | 93.0  (92.7-93.3) | 629  (465-847) | 6512  (4820-9034) | 0.6  (0.6-0.6) |
| NFP | 92.3  (91.8-92.6) | 462  (340-622) | 27746  (20660-37117) | 0.9  (0.9-0.9) | 84.0  (83.3-84.5) | 420  (308-568) | 17737  (13056-23738) | 1.0  (1.0-1.1) |
| Male STI | 93.9  (93.7-94.2) | 13610  (10837-17453) | 18637  (14630-24037) | 18.3  (17.3-18.8) | 88.0  (87.6-88.5) | 12753  (10127-16389) | 11012  (8660-14100) | 20.0  (19.3-20.6) |
| LDTD | 1.0  (0.6-1.6) | 2  (1-4) | 1087634  (822953-1446564) | 0.2  (0.1-0.3) | 0.1  (0.1-0.1) | 0  (0-0) | 1086186  (821942-1445344) | 0.0  (0.0-0.0) |
| General males | 0.1  (0.1-0.2) | 4  (3-6) | 1644421  (1271256-2059921) | 0.4  (0.4-0.5) | 0.1  (0.1-0.1) | 3  (2-4) | 1644245  (1271236-2059572) | 0.7  (0.7-0.7) |
| Pregnant women | 72.0  (63.2-76.4) | 468  (304-679) | 230906  (172452-311344) | 7.4  (6.5-8.2) | 0.1  (0.1-0.4) | 1  (0-4) | 218955  (163682-291728) | 0.0  (0.0-0.1) |
| General females | 0.1  (0.1-0.2) | 29  (19-47) | 218044  (163895-295301) | 0.5  (0.4-0.5) | 0.1  (0.1-0.1) | 22  (16-30) | 217960  (163878-294885) | 0.7  (0.7-0.7) |
| Overall | 79.7* (78.2-80.9) | 115337  (86092-155278) | 12257  (9542-16012) | 100 | 76.2*  (74.8-77.4) | 110290  (82346-148438) | 6446  (4979-8400) | 100 |

IQR: interquartile range. * represents the timely diagnosis rate among total annual new infections.

MSM: men who have sex with men; PWID: people who inject drugs; FSW: female sex workers; NMP: HIV-negative male of serodiscordant couples; NFP: HIV-negative female of serodiscordant couples; Male STI: male sexual transmission infections clinic attendees; LDTD: long-distancing truck drivers.

**Continued from Table S12**

| **Population** | **Optimized strategy with five-fold the baseline spending** | | | | **Optimized strategy with ten-fold the baseline spending** | | | | |
| --- | --- | --- | --- | --- | --- | --- | --- | --- | --- |
|  | **Optimized testing rate (%)**  **(median,**  **IQR)** | **Diagnoses in new infection**  **(median,**  **IQR)** | **Cost of detecting one HIV case (USD)**  **(median,**  **IQR)** | **Allocation of HIV resources (%)** | **Optimized testing rate (%)**  **(median,**  **IQR)** | **Diagnoses in new infection**  **(median,**  **IQR)** | **Cost of detecting one HIV case (USD)**  **(median,**  **IQR)** | **Allocation of HIV resources (%)** | |
| Transgender women | 98.8  (98.8-98.9) | 11598  (8858-15287) | 4630  (3495-5953) | 0.4  (0.4-0.4) | 98.9  (98.8-99.0) | 11614  (8858-15294) | 4806  (3642-6400) | | 0.2  (0.2-0.2) |
| High-risk MSM | 98.9  (98.8-99.0) | 31594  (24009-41797) | 7945  (6056-10119) | 1.8  (1.7-1.9) | 98.9  (98.8-99.0) | 31623  (24009-41803) | 7967  (6069-10465) | | 0.9  (0.9-0.9) |
| Money boys | 98.0  (97.0-98.6) | 5622  (4302-7463) | 26722  (17985-38485) | 1.1  (0.7-1.5) | 98.5  (97.5-98.9) | 5711  (4328-7483) | 34290  (21006-49197) | | 0.7  (0.4-1.0) |
| Low-risk MSM | 98.3  (98.2-98.5) | 27897  (20708-37596) | 35106  (26668-45597) | 6.9  (6.2-7.5) | 98.8  (98.3-98.9) | 28092  (20747-37773) | 44519  (32085-60781) | | 4.6  (3.6-5.3) |
| PWID | 98.6  (98.4-98.8) | 22280  (17117-29243) | 14770  (11089-19054) | 2.3  (2.0-2.8) | 98.8  (98.3-99.0) | 22384  (17114-29278) | 15992  (11124-21995) | | 1.3  (1.0-1.5) |
| High-risk FSW | 92.8  (89.1-95.0) | 1228  (915-1755) | 91888  (64548-128956) | 0.8  (0.6-1.1) | 95.9  (86.2-97.4) | 1323  (885-1801) | 140124  (66036-246764) | | 0.7  (0.3-1.0) |
| Low-risk FSW | 91.5  (89.2-92.5) | 1449  (1079-2014) | 152536  (113850-204256) | 1.6  (1.3-1.9) | 95.3  (87.6-96.2) | 1547  (1059-2095) | 246829  (125962-396537) | | 1.4  (0.6-1.9) |
| NMP | 92.8  (89.3-97.1) | 604  (448-881) | 7344  (4789-12528) | 0.0  (0.0-0.1) | 95.8  (88.2-98.6) | 647  (442-895) | 10120  (4965-24138) | | 0.0  (0.0-0.1) |
| NFP | 92.5  (88.0-96.9) | 440  (326-651) | 31990  (20820-57123) | 0.1  (0.1-0.2) | 95.1  (87.3-98.3) | 476  (323-661) | 42163  (21515-104299) | | 0.1  (0.0-0.2) |
| Male STI | 98.0  (97.7-98.5) | 14159  (11302-18244) | 50449  (38053-69029) | 5.2  (4.5-6.6) | 98.7  (98.7-98.9) | 14305  (11409-18314) | 78247  (60341-101258) | | 4.1  (3.9-4.5) |
| LDTD | 32.2  (22.9-60.2) | 48  (36-167) | 1238619  (942897-1628888) | 0.6  (0.4-1.2) | 69.9  (24.7-89.4) | 148  (39-248) | 1700553  (1105044-3078497) | | 0.9  (0.2-1.8) |
| General males | 24.2  (21.4-26.0) | 644  (510-1026) | 1809371  (1391636-2272269) | 9.2  (8.2-10.2) | 66.9  (63.3-69.3) | 2009  (1507-2733) | 2669436  (2026986-3477867) | | 19.6  (18.0-21.3) |
| Pregnant women | 96.7  (95.6-97.3) | 621  (459-864) | 327462  (240662-442509) | 1.5  (1.3-1.6) | 97.5  (97.5-97.6) | 633  (469-867) | 381159  (281035-507633) | | 0.9  (0.8-0.9) |
| General females | 81.5  (80.9-81.9) | 17541  (13158-24227) | 536494  (406040-706116) | 68.4  (65.7-69.8) | 90.3  (89.9-90.7) | 19577  (14620-26828) | 883367  (670428-1171756) | | 64.5  (59.8-67.6) |
| Overall | 94.1*  (93.7-94.4) | 135650  (103197-181188) | 103636  (79239-133747) | 100 | 96.7*  (96.0-96.9) | 140035  (105756-185929) | 199485  (151555-257935) | | 100 |

IQR: interquartile range. * represents the timely diagnosis rate among total annual new infections.

MSM: men who have sex with men; PWID: people who inject drugs; FSW: female sex workers; NMP: HIV-negative male of serodiscordant couples; NFP: HIV-negative female of serodiscordant couples; Male STI: male sexual transmission infections clinic attendees; LDTD: long-distancing truck drivers.
